# Tungsten Oxide Mediated Quasi-van der Waals Epitaxy of WS_2_ on Sapphire

**DOI:** 10.1021/acsnano.2c09754

**Published:** 2023-03-08

**Authors:** Assael Cohen, Pranab K. Mohapatra, Simon Hettler, Avinash Patsha, K. V. L. V. Narayanachari, Pini Shekhter, John Cavin, James M. Rondinelli, Michael Bedzyk, Oswaldo Dieguez, Raul Arenal, Ariel Ismach

**Affiliations:** † Department of Materials Science and Engineering, Tel Aviv University, Ramat Aviv, Tel Aviv 6997801, Israel; ‡ Laboratorio de Microscopías Avanzadas (LMA), Universidad de Zaragoza, 50018 Zaragoza, Spain; § Instituto de Nanociencia y Materiales de Aragón (INMA), CSIC−Universidad de Zaragoza, 50009 Zaragoza, Spain; ∥ Center for Nanoscience and Nanotechnology, Tel Aviv University, Tel Aviv 6997801, Israel; ^⊥^Department of Materials Science and Engineering and ^∇^Department of Physics and Astronomy, Northwestern University, Evanston, Illinois 60208, United States; ¶ ARAID Foundation, 50018 Zaragoza, Spain

**Keywords:** metal organic chemical vapor deposition, quasi-van der Waals epitaxy, interface, surface modification, transition metal dichalcogenides, tungsten trioxide

## Abstract

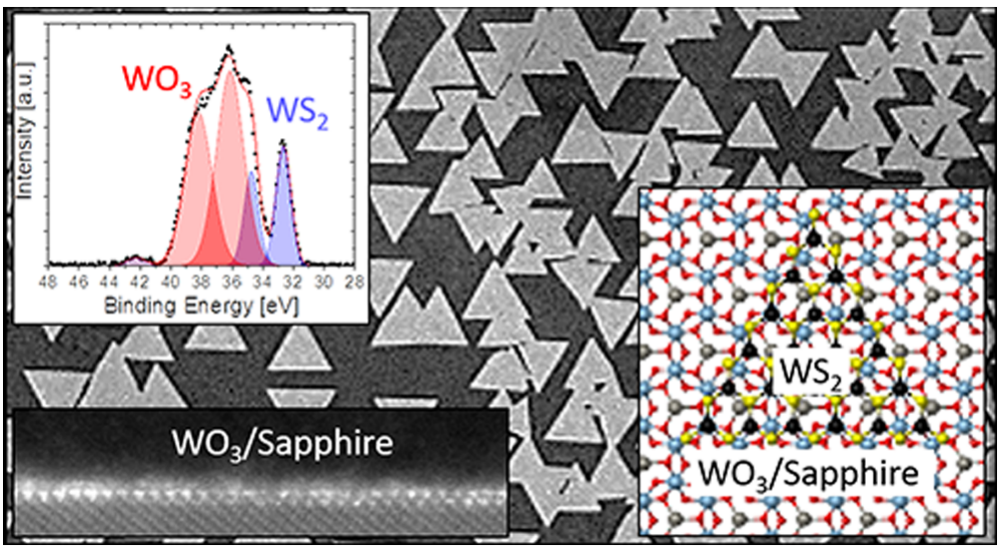

Conventional epitaxy plays a crucial role in current state-of-the art semiconductor technology, as it provides a path for accurate control at the atomic scale of thin films and nanostructures, to be used as the building blocks in nanoelectronics, optoelectronics, sensors, etc. Four decades ago, the terms “van der Waals” (vdW) and “quasi-vdW (Q-vdW) epitaxy” were coined to explain the oriented growth of vdW layers on 2D and 3D substrates, respectively. The major difference with conventional epitaxy is the weaker interaction between the epi-layer and the epi-substrates. Indeed, research on Q-vdW epitaxial growth of transition metal dichalcogenides (TMDCs) has been intense, with oriented growth of atomically thin semiconductors on sapphire being one of the most studied systems. Nonetheless, there are some striking and not yet understood differences in the literature regarding the orientation registry between the epi-layers and epi-substrate and the interface chemistry. Here we study the growth of WS_2_ via a sequential exposure of the metal and the chalcogen precursors in a metal–organic chemical vapor deposition (MOCVD) system, introducing a metal-seeding step prior to the growth. The ability to control the delivery of the precursor made it possible to study the formation of a continuous and apparently ordered WO_3_ mono- or few-layer at the surface of a *c*-plane sapphire. Such an interfacial layer is shown to strongly influence the subsequent quasi-vdW epitaxial growth of the atomically thin semiconductor layers on sapphire. Hence, here we elucidate an epitaxial growth mechanism and demonstrate the robustness of the metal-seeding approach for the oriented formation of other TMDC layers. This work may enable the rational design of vdW and quasi-vdW epitaxial growth on different material systems.

## Introduction

2D materials in general and atomically thin semiconductors in particular, such as the transition metal dichalcogenides (TMDC) family, are at the center of the scientific and technological communities’ interest due to their unique physical and chemical properties and potential applications.^1−3^ However, as is often the case for novel materials, one of the prerequisites for their successful integration into potential technologies is the ability to grow them with the desired structure (number of layers, phase, chemical composition, etc.) and thus properties.^3^ Chemical vapor deposition (CVD) based methodologies, which are widely employed in industry for the formation of a large variety of metallic, semiconductor, and insulator thin films,^4^ are also considered very promising for the growth of TMDCs.^1,5−8^ In such vapor phase growth methodologies, the precursor compounds’ type represents one of the most important factors in the growth, as the kinetics of the process is highly influenced by this choice. Hence, despite great advances at the synthetic front with the most commonly used metal oxide precursors, their low vapor pressures and proximity to the growth substrate limit their implementation for growth mechanistic studies and the large-scale growth of TMDCs.^1,4,7−9^ Therefore, the use of volatile precursors, for both the metal and chalcogen atoms, is gaining interest, due to a greater suitability for CVD based methodologies, with metal–organics and metal-halides as the main sources.^6−8,10,11^ Once the low-pressure metal-oxide precursors are replaced with the volatile compounds, the latter can be placed outside the reactor and delivered via a carrier gas, allowing for independent control of the flux of each precursor (metal and chalcogen) to the growth area. The ability to do so is shown to be critical for basic growth mechanism studies and improvement of the grown layer.^8,10,12,13^

The ability to independently control the delivery of the different precursors to the substrate, using volatile metal (W(CO)_6_) and chalcogen (DTBS or H_2_S) precursors in an MOCVD system, is crucial for achieving better control and understanding of the growth process. In our previous work, the high nucleation density usually obtained in MOCVD processes^6,8,10,12−14^ was reduced using a pulsed flow of the W(CO)_6_ precursor. This together with the flow of low amounts of water vapor caused the re-evaporation of carbon contaminants (which act as nucleation sites), as well as small and defective WS_2_ domains, leading to highly crystalline TMDC domains and films.^10,15^ Hence, the implementation of a sequential precursor delivery to the substrate led to better control over the nucleation and growth, as reflected from the improved optical properties.^10,15^ The crystallinity of the film might be further improved by increasing the domain size and via vdW or Q-vdW epitaxial growth.

The basic requirement to achieve conventional epitaxial growth (a 3D crystal on a 3D substrate) is a strong chemical bond (covalent, ionic, or metallic) between the epi-layer and the substrate. Four decades ago, Koma and co-workers coined the term VdW and quasi-vdW epitaxy to explain the oriented growth of layered compounds on 2D and 3D substrates, respectively.^16^ This pioneering work was originally obtained using molecular beam epitaxy (MBE) and metal–organic MBE (MO-MBE) techniques.^16^ The renewed interest in 2D materials in general and their growth in particular triggered the community to search for more accessible processes based on CVD methodologies. Indeed, vdW and quasi-vdW epitaxial growth has been extensively reported in the past two decades, with graphene,^3,17^ h-BN,^18,19^ TMDCs,^11,12,17,19−29^ and others.^30^ VdW epitaxy of TMDCs was demonstrated on graphene,^17^ h-BN,^18,19^ and other TMDCs,^27^ while quasi-vdW was obtained on GaN,^28^ sapphire,^12,20,21,23,25,31−33^ and gold,^26^ with the growth on single-crystal alumina being one of the most studied systems. In the particular cases of vdW and quasi-vdW systems, the epitaxial growth with large lattice mismatch was demonstrated possible,^16^ presumably due to the weak interaction between the layers (vdW epitaxy) and the layer-substrate (quasi-vdW epitaxy). Therefore, such oriented growth by itself cannot ensure the absence of domain boundaries.^34^ Hence, in the ideal case, a low nucleation density and vdW epitaxial growth together are desired.

As mentioned above, the epitaxial growth of TMDCs on sapphire substrates was widely reported. In general, a *c*-plane α-Al_2_O_3_ is used,^12,21,23,25,31−33^ however *a*-plane was also studied.^20^ Such Q-vdW epi-growth was achieved using the more common metal-oxide (M–O–CVD) precursors (usually MeO_3_, where Me = W or Mo), metal carbonyls (MOCVD), and metal halides (MHCVD). Despite the observed oriented growth in all these reports, there are some striking and not yet understood differences. The orientation registry between the TMDC and the *c*-plane sapphire was reported to be [11–20]//[11–20]^12,21,25,32,33,35−37^ or [11–20]//[10–10],^23,24,31,33,36^ see Table S1. The chalcogen to metal-oxide flow ratio was reported to play a crucial role on the orientation of the domain growth. For example, Suenaga et al. studied the effect of the sulfur concentration in the growth of MoS_2_, concluding that aligned growth along [11–20] and [1–100] is achieved with reducing amounts of sulfur.^33^ In contrast, Lai et al. reported that a high S/MoO_*x*_ ratio results in [11–20]//[10–10] aligned growth, while lower ratios [11–20]//[11–20] and further reduction ended in nonoriented domain growth.^24^ In addition to the chalcogen/metal precursor flow ratio, Hwang et al. studied the influence of the hydrogen flow in epitaxial growth and concluded that it plays a vital role by enhancing the nucleation at the step edges and thus resulting in aligned growth.^31^ It is important to note that the more common M–O precursors were used in these reports, which, as mentioned above, are challenging to control and monitor their delivery during growth. The inherent anisotropy of the *c*-plane alumina surface due to the presence of atomic steps may also influence the growth as well. Indeed, step-edge guided growth was demonstrated, using metal oxide and metal organic precursors.^25,32^ Atomic steps on *c*-plane sapphire not only act as nucleation points but also may break the degeneracy of nucleation energy for the antiparallel domains, thus leading to unidirectional growth when cutting the crystal toward the *a* axis.^23^ Koma and co-workers suggested that, in order for quasi-van der Waals epitaxial growth to occur, the 3D surface should be passivated to preserve the vdW nature of the grown layer.^16^ The groups of Robinson and Redwing, in their extensive work on Q-vdW epitaxy of TMDCs on *c*-plane sapphire via MOCVD, proposed that a chalcogen (Se) terminated α-Al_2_O_3_ surface is formed, promoting the oriented growth.^12,35,37^ In these reports, the Se interfacial layer was characterized using cross section aberration corrected HAADF-STEM (contrast-based analysis), EDS, and modeling using DFT calculations. Based on all these reports, it seems there are more than a single possible epitaxial growth mechanism. Hence, despite the great advances in the field, further research is still needed to elucidate the parameters dictating the oriented growth, with the chemistry at the interface, in our opinion, being one of the most important ones.

Here, we study the growth of WS_2_ via a sequential exposure of the metal and the chalcogen precursors using a metal–organic chemical vapor deposition (MOCVD) system. The ability to control the delivery of each precursor separately enabled us to study the formation of an ordered WO_3_ layer on top of the *c*-plane sapphire surface. The atomically thin tungsten oxide layer is characterized using X-ray photoelectron spectroscopy (XPS), time-of-flight secondary ion mass spectroscopy (TOF-SIMS), in-plane grazing-incidence X-ray diffraction, X-ray fluorescence (XRF), high-resolution scanning transmission electron microscopy (HRSTEM), energy dispersive X-ray spectroscopy (EDS) STEM, electron energy-loss spectroscopy (EELS) STEM, and density functional theory (DFT) calculations. In our case, the formation of such an interfacial layer is shown to be crucial for the quasi-vdW epitaxial growth of the TMDC layers on sapphire. The elucidation of the interfacial layer needed to obtain such oriented growth might lead to the possibility of rational expansion of epitaxial growth on other material systems (epi-layer and -substrate).

## Results and Discussion

### Quasi-van der Waals Epitaxial Growth

In general, heteroepitaxial growth is highly influenced by several parameters such as the crystal orientation and reconstruction of the surface, surface chemical modification, miscut orientation, and the process parameters (temperature, pressure, gas composition and flow, etc.). Therefore, in an attempt to improve the crystallinity via epitaxial (Q-vdW) growth, significant efforts were invested in our lab on the sapphire surface preparation (including thermal and chalcogen pretreatments) as well as growth process parameters optimization. At this stage, however, we could only obtain sporadic, nonuniform, and uncontrolled Q-vdW epitaxial growth, with very low yield.

**Figure 1:**
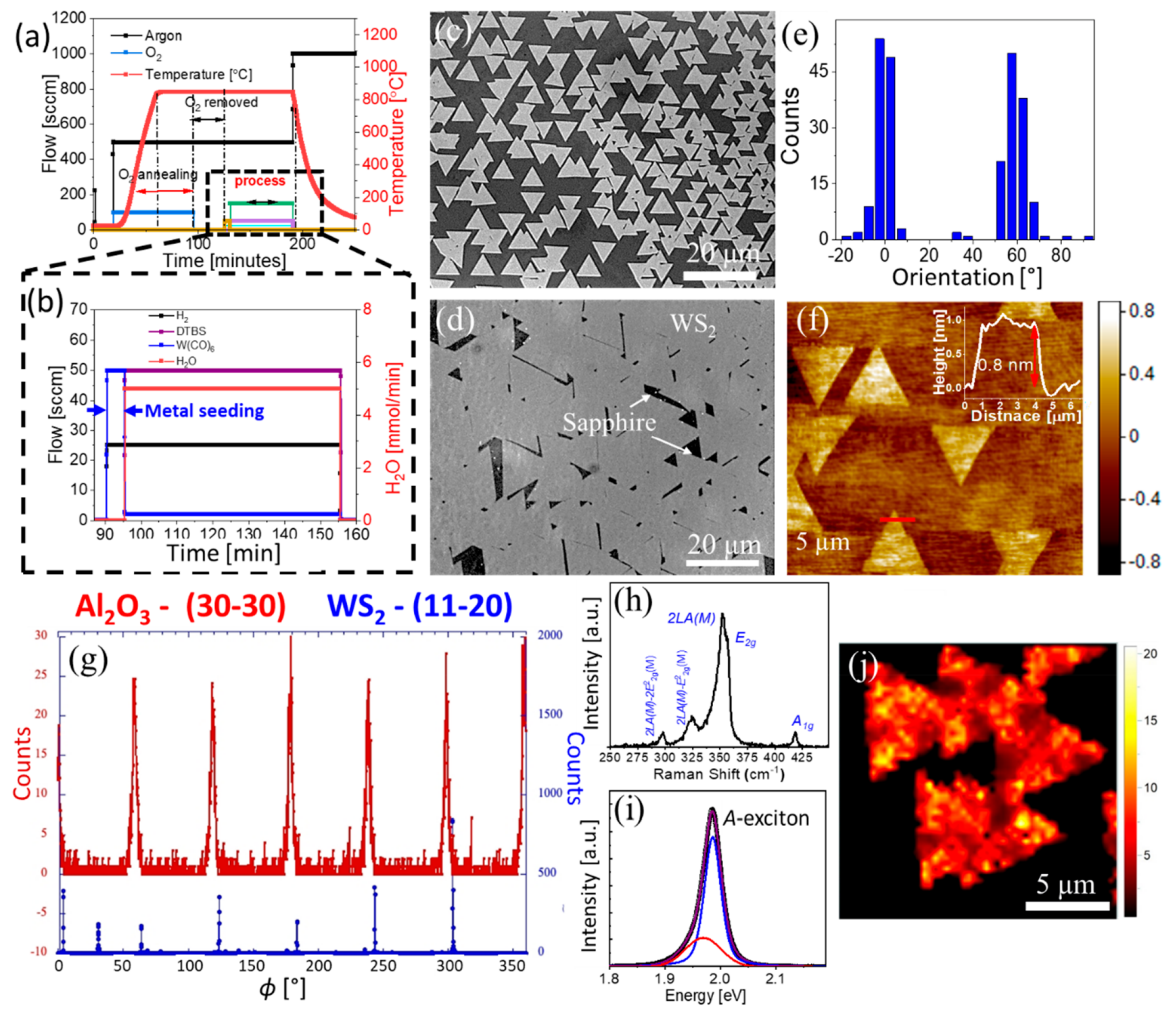
Metal-seeding quasi-vdW epitaxial growth of WS_2_. (a,b) Flow scheme showing the metal-seeding process. (c,d) Optical microscope images showing different degree of epitaxial growth coverage. (e) Domain orientation distribution analysis. (f) AFM image showing the oriented growth with the crystallographic directions of the sapphire surface. The inset shows a cross section AFM measurement along the red line. (g) In-plane grazing incidence X-ray diffraction results showing azimuthal ϕ-scans at fixed 2θ scattering angles for the Al_2_O_3_(30–30), blue, and WS_2_(11–20), red, Bragg conditions. (h) Raman spectra of the domains showing the characteristic WS_2_ modes. (i) PL spectra of the domains with a full width half-maximum (fwhm) of 28 meV. The deconvolution of the spectra shows the contributions of the A exciton (blue) and trion (red). (j) Raman mapping using the peak intensity ratio *I*(2LA(*M*))/*I*(*A*_1g_).

Taking advantage of the use of volatile precursors (Figure S1), we aimed to study the growth behavior as a function of precursor delivery sequence. In this regard, a metal-seeding step, in which the substrate is exposed only to the metal–carbonyl (W(CO)_6_) precursor, is implemented as schematically depicted in Figures 1(a,b) and S2. The addition of such a metal-seeding step had the immediate effect of causing consistent growth of epitaxial Q-vdW. The optical micrographs (Figures 1(c,d) and S3) of as-grown WS_2_ samples show the aligned growth of WS_2_ domains on sapphire substrate, with preferred relative orientation along 0° and 60° Figure 1(e). A typical atomic force microscopy (AFM) image of such samples is shown in Figure 1(f). The line profile drawn across a single triangular domain (indicated by solid red line in the inset of Figure 1(f)) shows the thickness of ∼0.8 nm, confirming the monolayer nature of the WS_2_ domain. In-plane grazing incidence XRD was used to analyze the crystallographic registry between the WS_2_ domains and the *c*-plane sapphire substrate, see Experimental Section for details. Several in-plane grazing-incidence X-ray diffraction peaks were collected to show that the WS_2_ formed single crystals with orientational epitaxy to the substrate lattice. The results shown in Figure 1(g) demonstrate the WS_2_ [11–20] 2D crystal direction was parallel to the α-Al_2_O_3_ [30–30] direction and exhibited 6-fold symmetry. The ϕ angle is related to the Bragg angle which will differ because the *d*-spacings of the film and subtract reflections are different. The in-plane WS_2_ hexagonal lattice constant was determined to be a = 3.156 Å, which is 1.1% smaller than that of bulk WS_2_ (*a* = 3.191 Å). The azimuthal ϕ-scan widths for the {11–20}WS_2_ peaks indicated a mosaic spread of 3.5°, see Figures S4–S6 and Experimental Section for details. Raman spectrum, Figure 1(h), of the as-grown domains shows the strong convoluted peak of 2*LA*(*M*) (∼350 cm^–1^) and *E*_2g_ (∼356 cm^–1^) modes, and low intensity *A*_1g_ (∼418 cm^–1^) mode, corresponding to 2H phase WS_2_.^38^ The extra low intense modes around ∼297 and ∼325 cm^–1^ correspond to 2*LA*(*M*) – *E*^2^_2g_(M) and 2*LA*(*M*) – 2*E*^2^_2g_(*M*), respectively. The peak intensity ratio *I*(2LA(*M*))/*I*(*A*_1g_) is found to be >4, confirming the monolayer nature of WS_2_ domains.^38^ The monolayer nature of as-grown WS_2_ domains is further revealed by the intense photoluminescence (PL) peak around 1.99 eV, Figure 1(i), corresponding to the *A*-exciton emission. The low energy broad peak ∼1.97 eV corresponds to the charged exciton (trion) due to intrinsic doping.^39^ A typical Raman spectroscopic map with the intensity ratio of 2*LA*(*M*) and *A*_1g_ modes [*I*(2LA(*M*))/*I*(*A*_1g_) > 4], Figure 1(j), reveals that the aligned WS_2_ domains are single-layers.

### Surface and Interface Characterization

**Figure 2:**
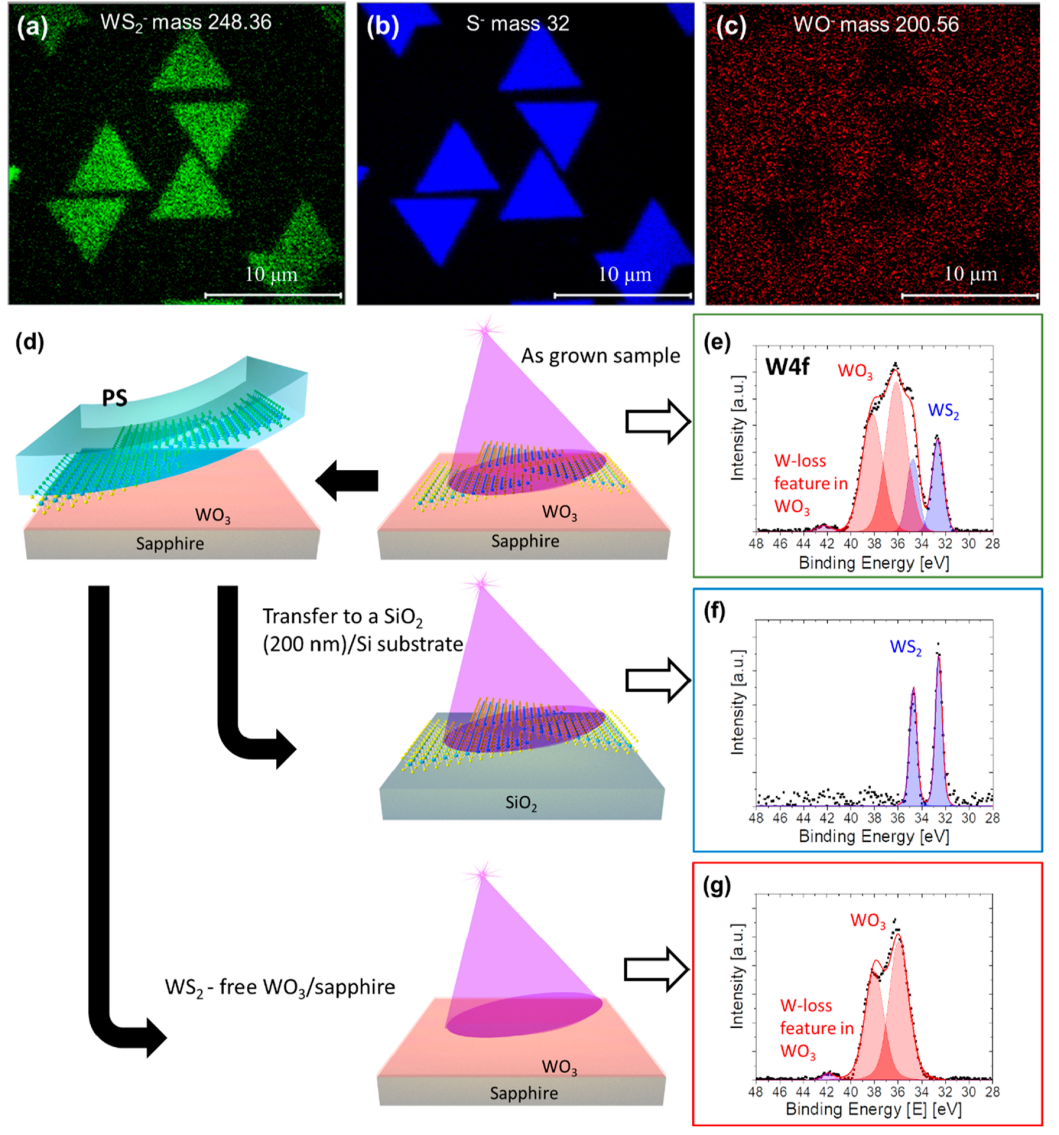
Surface chemical characterization. (a–c) TOF-SIMS maps of masses corresponding to the WS_2_^–^, S^–^, and WO^–^ ions, respectively, on an as-grown sample, showing that the W–O is present all over the surface of sapphire. (d) Schematic representation of the PS-based transfer of WS_2_ domains on to SiO_2_/Si substrate (indicated by arrows on left side), and the samples considered for XPS study (on right side). The area under analysis on each sample (right side) is indicated by purple conical structure representing the X-ray beam. XPS spectra of W 4f recorded from the as-grown WS_2_ sample on sapphire (e), from the transferred WS_2_ on SiO_2_/Si (f), and from the WS_2_-free sapphire substrate (g). The doublet arising from W^(+4)^S_2_ (blue) at 33.6 eV is observable only on as-grown (e) and transferred WS_2_ samples (f), whereas on WS_2_-free sapphire substrate (g), solely the doublet arising from W^(+6)^O_3_ (red) at 36.1 eV which corresponds to the WO_3_ phase is observed.

Chemical analysis and imaging with high lateral resolution (∼200 nm) using time-of-flight secondary ion mass spectroscopy (TOF-SIMS)^40^ was performed. Figures 2(a–c) show the mapping of masses corresponding to WS_2_^–^, S^–^, and WO^–^ negative ions, respectively. As expected, the WS_2_^–^, Figure 2(a), and S^–^, Figure 2(b), ions are present only on the WS_2_ triangular domains. Interestingly, the mapping shows that a W–O phase is present all over the surface, Figure 2(c). XPS was employed to further elucidate the tungsten oxide phase. Figure 2(d) depicts the fabrication scheme used for separating the WS_2_ layer from the WO_3_ layer. Figures 2(e–g) show the XPS W 4f ^5^/_2_ and ^7^/_2_ doublet for each stage of this process, starting with the as-grown epi-WS_2_ sample on sapphire, Figure 2(e), where there are two doublets arising from the W^(+4)^S_2_ (blue) and W^(+6)^O_3_ (red) bonds with the ^7^/_2_ peak binding energy at 32.6 and 36.1 eV, respectively.^41^ Images obtained by mapping with the W–S and W–O bond energies, 33.2 and 36.4 eV, respectively, are shown in Figures S7(a,b). While the W–S signal appears only on the WS_2_ domains, as expected, the WO_3_ can be seen all over the surface. The slight intensity enhancement, Figure S7(b), that can be observed for the W–O signal in the same position where W–S can also be seen is attributed to an overlap between the two peaks and the background enhancement following the W–S peak, but not to a thickness change of the oxide film. Following the characterization of the as-grown sample, Figure 2(e), the WS_2_ domains were transferred from the growth substrate (sapphire) to a SiO_2_/Si substrate using polystyrene (PS) based transfer process, see Experimental Section for details. The transferred WS_2_ film on a SiO_2_/Si and the WS_2_-free sapphire substrate were further characterized by XPS as well, as shown in Figures 2(f) and (g), respectively. In the first, only the WS_2_ doublet is observed, Figure 2(f), suggesting only the latter is transferred. Interestingly, on the WS_2_-free sapphire surface, the WO_3_ peaks were still detected, Figure 2(g). Hence, it can be concluded that a continuous and strongly bonded WO_3_ phase is formed on the entire sapphire surface when Q-vdW epitaxy is achieved.

**Figure 3:**
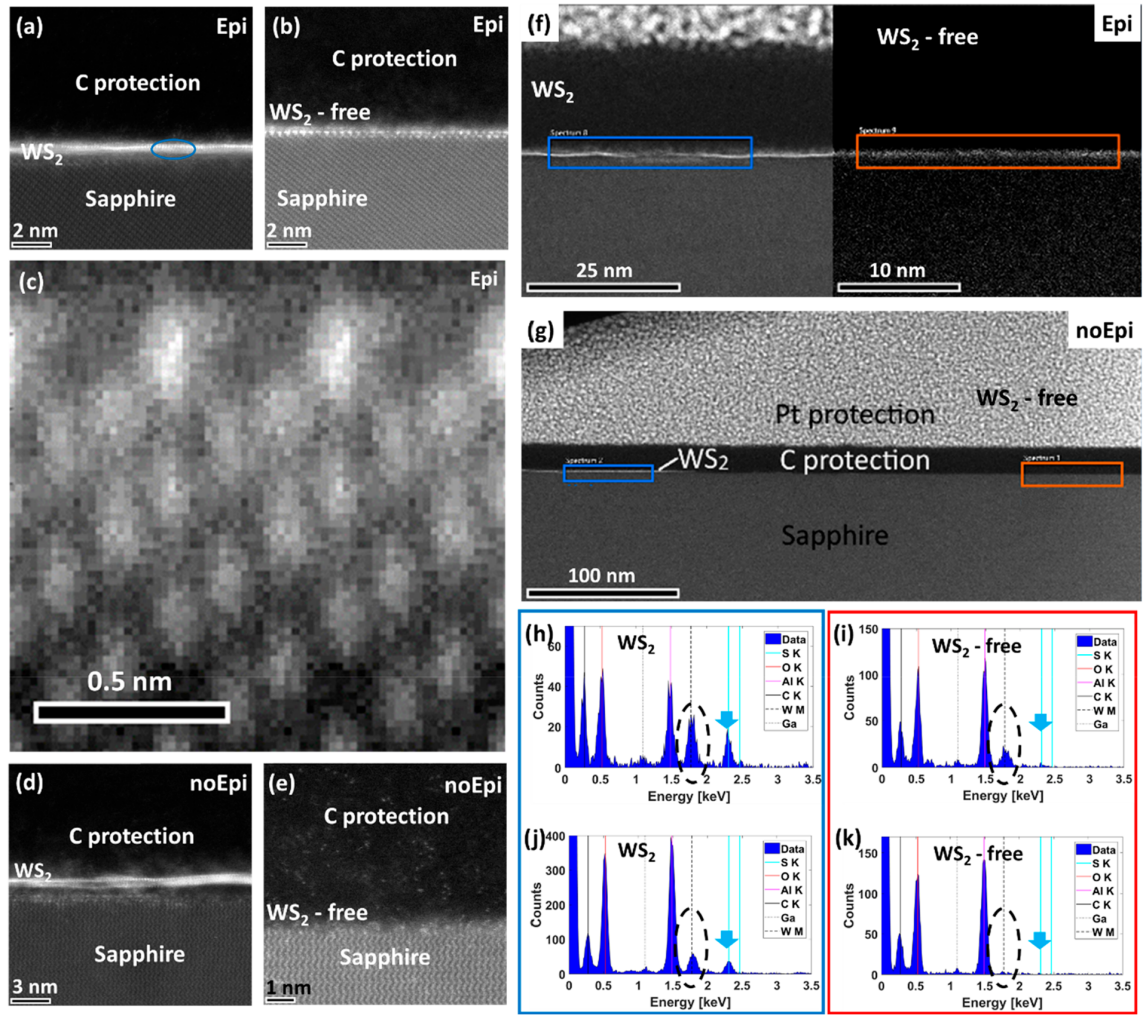
Transmission electron microscope characterization. HAADF-STEM images of (a–c) Epi and (d,e) noEpi sample showing a region (a,d) with and (b,c,e) without WS_2_ grown on the surface. (c) Atomic arrangement at the interface, which was obtained from averaging several unit cells of a high-resolution image (Figure S9). WS_2_ is observed directly at the sapphire surface in (a) and (d). The sapphire surface terminates in an ordered bright structure in (b) and (c), which is absent in (e). (f,g) Low magnification images of the Epi grown (f) and noEpi (g) samples showing the WS_2_ (left) and WS_2_-free zones (right). STEM-EDS analyses of the (h,i) Epi and (j,k) noEpi lamellae. In both samples, an area with (h,j) and without (i,k) WS_2_ grown on the surface is compared, which are marked by blue and orange in (f) and (g), respectively. WS_2_ layer is also visible in left image of (f), while the right part only shows brighter surface termination of the sapphire. EDS spectra reveal the presence of C from protection layer, Al and O from sapphire substrate, and Ga remainders from lamella fabrication. S (light blue arrow) is only found in WS_2_ layer (h,j). W peaks (marked by black ellipses) are observed in the (h,j) WS_2_ layers and also (i) at the WS_2_-free surface of the sapphire in the Epi sample, while being missing (k) at the surface of the noEpi sample.

To further study the nature of the WO_3_ sapphire termination layer, cross-sectional TEM lamellae were prepared and characterized using high resolution scanning transmission electron microscopy (HRSTEM), see the Experimental Section for details. Two types of samples were investigated in detail, epitaxially grown WS_2_ via the metal-seeding approach described above, named Epi, and nonepitaxial WS_2_ domains grown via the growth-etch methodology,^10^ noEpi. In both cases, cross-section lamellae were prepared by focused ion beam (FIB) in WS_2_ and WS_2_-free areas, see Experimental Section and SI for details. Figures 3(a–e) show four high-angle annular dark field (HAADF)-STEM images of different areas of the Epi and noEpi lamellae. The carbon protection layer (top) and sapphire substrate (bottom) are visible in all images. In the case of the Epi sample, (a), a monolayer of WS_2_ is observed on top of the sapphire substrate. The distance between bright atomic W columns is measured to be 0.16 nm corresponding with the (200) direction of the WS_2_ lattice (blue ellipse in Figure 3(a)). At the sapphire surface, atomic rows with an intermediate intensity between W and sapphire can be observed. Similar rows are present in areas without WS_2_ as clearly visible in Figure 3(b). This interface can be composed of a single layer or of few atomic layers. Additional high-resolution STEM images are shown in Figures S8–S10. The atomic arrangement at the interface, which was obtained from averaging several unit cells of a high-resolution image (Figure S9), is shown in Figure 3(c). In the noEpi sample, a bright atomic row, somewhat less intense (lower density), is observed underneath the TMDC domain, Figure 3(d), but absent on the WS_2_-free areas, Figure 3(e). STEM-energy dispersive X-ray spectroscopy (EDS) analyses of the interface region of the two samples, Figures 3(f,g), reveal the presence of various elements. C, Al, and O as well as Ga can be attributed to the C protection layer, the sapphire substrate, and impurities from the FIB TEM lamella fabrication process, respectively. The blue and red rectangles in Figures 3(f,g) are the EDS scan areas for the WS_2_ and WS_2_-free regions, respectively. Sulfur is only present in areas with WS_2_ grown on the surface, as indicated by a light blue arrow in Figure 3(h), epi, and Figure 3(j), noEpi samples and absent in the WS_2_-free areas, Figures 3(i,k), in both samples. The tungsten peak position is emphasized by the black dashed ellipses. As expected, it is found in the WS_2_ regions, Figures 3(h,j). Analyzing the orange rectangles corresponding to the WS_2_-free areas marked on Figures 3(f,g), tungsten is also found on the Epi sample, Figure 3(i), but absent on the noEpi sample, Figure 3(k). Thus, EDS analysis suggests the presence of a W-terminated sapphire surface in the Epi sample. This analytical finding agrees with the bright contrast at the sapphire surface seen in imaging, Figures 3(b,e), and can be explained by the presence of heavy W atoms, which increase the intensity in the HAADF-STEM with the atomic number (∼Z^1.7^). The intensity difference suggests that the W atom density at the interface is lower than in the WS_2_ layer.

In order to study the effect of the metal seeding exposure and the growth time, several samples were prepared and analyzed, Figures S11–S13. Metal seeding of 5 min and growth (addition of DTBS) for 1 min resulted in some nanoparticles as seen in Figure S11(a), with no WS_2_ domains observed. Increasing the growth time to 5 min (metal seeding of 5 min as well) ended up in relatively small aligned WS_2_ domains and some nanoparticles on the substrate, as seen in the AFM images in Figures S11(b,c). Finally, the results for long metal precursor exposures of 30 min, with no DTBS, are shown in Figures S12–S13. Such extended metal seeding steps caused the formation of WO_*x*_ nanowires, preferentially aligned in 3-fold symmetry, following the one of the substrate lattice. Nanoparticles with comparable sizes of the nanowires (NWs), 30–50 nm, are observed as well, Figure S12. Cross section STEM analysis done on such samples clearly show the same W-based layer at the interface with the nanowire and on NW-free areas, Figures S13(a–c). EDS analysis of the same areas clearly show the presence of W on the sapphire surface, Figure S13(d). XPS characterization of such samples, Figure S14, shows a significant contribution from a W^5+^ doublet, probably arising from mixed tungsten oxide phases in the WO_*x*_ NWs. As mentioned above and in the Experimental Section, most of the growth experiments were carried out around 850 °C; however, metal-seeding-induced Q-vdW epitaxial growth was obtained at higher temperatures, up to 950 °C (not shown), suggesting the WO_3_ termination is stable up to the above temperature, and maybe more.

**Figure 4:**
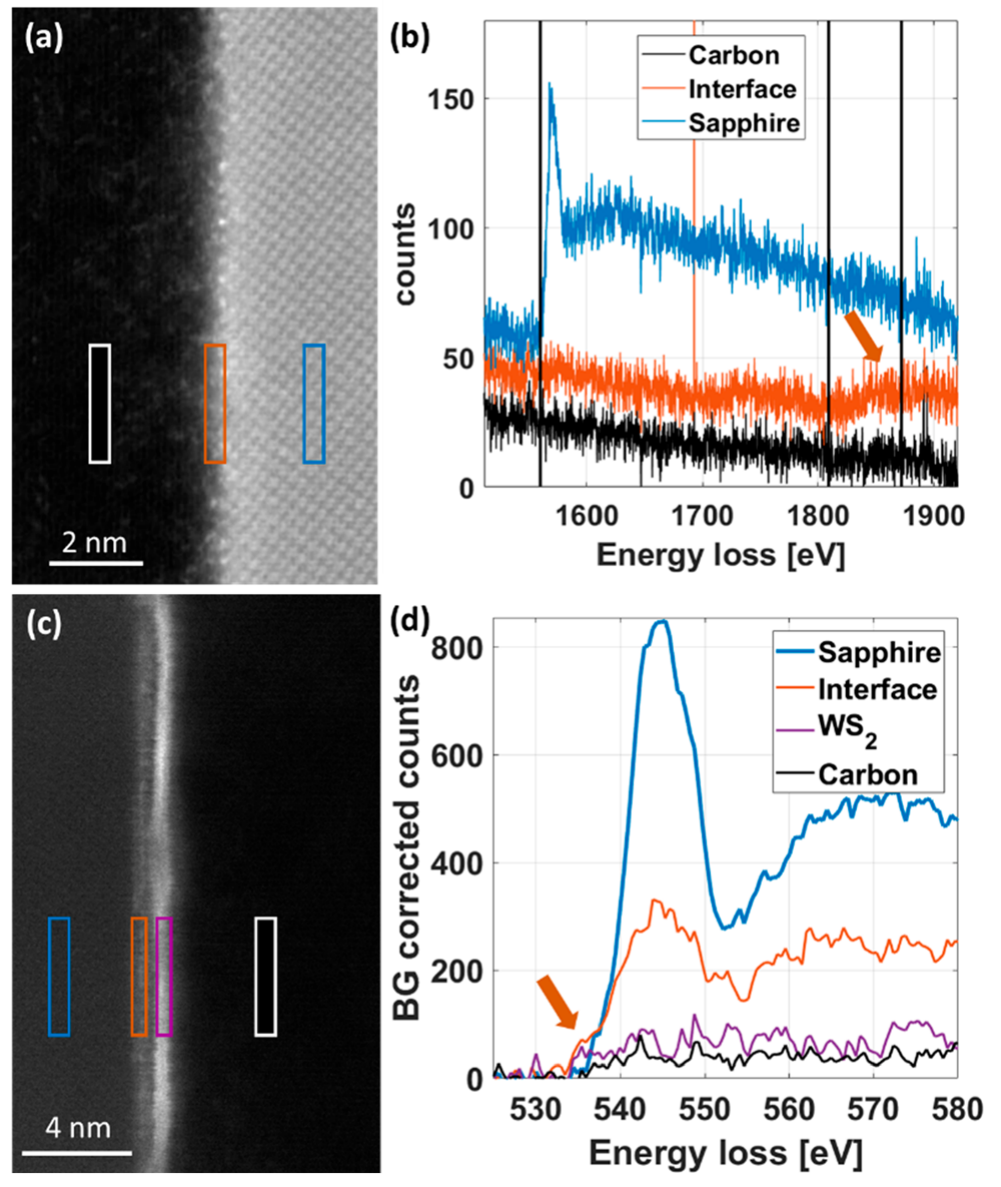
Core-loss EELS analysis of sapphire surface. (a) STEM-DF image shows sapphire substrate in WS_2_-free region and interface to C coating. (b) EEL spectra of carbon, interface, and sapphire region as marked in (a). Al–K edge is clearly resolved in the sapphire region and a slight increase in intensity at W–M edge position is observed in the interface region. (c) STEM-DF image of sapphire, carbon protection, and interface region. (d) O–K edge onset shifts to lower energies (red arrow) at the interface region, indicating a change in bonding properties.

To further investigate the differences between the Epi and noEpi samples’ interface, spatially resolved (SR)-EELS was performed in both core-loss and low-loss regions. Figure 4(a) shows the EELS analysis at higher energy losses of the sapphire surface in a region of the Epi sample, where no WS_2_ is observed. The spectrum obtained from the sapphire region clearly reveals the Al–K edge (blue curve in Figure 4(b)), which is strongly reduced in intensity at the interface (red curve). The slight increase in intensity above 1800 eV at the interface can be attributed to the W–M edge, marked by an orange arrow in Figure 4(b), confirming, once again, the presence of W atoms at the top surface in WS_2_-free regions.

Figures 4(c,d) show the analysis of the O–K edge at the interface region between the sapphire and a WS_2_ epi-grown layer. While a characteristic broad peak at 545 eV is observed in the sapphire substrate (blue curve in Figure 4(d)), the edge onset shifts to lower energies in the interface region (red curve and arrow). This shift is caused by a change in the bonding characteristics of the O atoms, which is attributed to the presence of W atoms. Indeed, SR-EELS analysis of noEpi samples and the sample with 30 min metal-seeding step shows that in the thicker, W-containing interface layer between sapphire and WS_2_ as well as in the WO_*x*_ NWs (see Figures S15–S17), the same shift of the O–K onset energy is observed. In the area analyzed in Figure S17, a non-negative matrix factorization (NNMF) of the EEL spectra results in three factors, which are attributed to sapphire, carbon containing material, and the interface region. A small peak at the position of the W–N_2,3_ edge is observed for the factor attributed to the interface. This indicates that the lower onset energy is linked to the presence of W atoms and serves as a reference for the analysis of the Epi sample (Figures 4(b–d)). EELS studies of tungsten oxides also show an early energy onset of the O–K edge.^42,43^ The core-loss EELS analysis therefore strongly indicates the formation of a WO_*x*_ interface layer at the sapphire surface. While this layer is occasionally formed also on the noEpi samples, the ordered and packed formation on the epitaxially grown samples is caused by the W precursor pregrowth seeding step, and it appears to be crucial for the WS_2_ oriented growth.

In addition to core-loss EELS analysis, acquisition of low-loss EEL spectra was performed as well. Figure S18 and S19 show exemplary results obtained from the Epi sample from a region with, Figure S18, and without, Figure S19, WS_2_ coating. In both cases, NNMF analysis was performed to disentangle the overlapping contributions from the different layers. The four factors found in the area with WS_2_ are displayed in Figure S18(b) and are assigned to contributions from the carbon protection layer, WS_2_, interface region, and sapphire. The different factors show varying positions of the bulk plasmon peak, which are determined by peak fitting.^44^ The peak energies of 23.2 and 25.9 eV for the WS_2_ and sapphire layers agree well with literature values.^45,46^ The plasmon energy of the interface layer (25.2 eV) is found in between the two values and resembles the one for WO_3_.^43^ To check consistency, raw spectra from four different regions are compared with the sum of individual NNMF contributions and are found to agree perfectly (not shown). The spatial distribution of carbon, interface, and sapphire agree very well with the expected distribution from imaging; only the WS_2_ distribution shows a minimum at the actual position of the corresponding layer, Figure S18(c). This is attributed to the strong scattering strength of the W atoms, which causes an overall decrease of intensity reaching the EEL spectrometer. Although beam damage could be minimized by scanning in a direction perpendicular to the interface, Figures S18(a) and S19(a), a minor oxidation of the WS_2_ layer may be observed and manifests itself in the decrease in intensity of the WS_2_ distribution map from top to bottom layer and a corresponding increase in the interface (WO_3_) map in Figure S18(c).

Figure S19 shows the analysis of a WS_2_-free region. Again, the rows with brighter contrast are found on top of the sapphire surface in the STEM-DF image, Figure S19(a). A NNMF analysis is also performed on this data set, which results in three factors assigned to carbon protection layer, interface, and sapphire substrate. Plasmon energy obtained for sapphire (26.1 eV) again agrees with literature,^45^ and the energy of 25.2 eV found for the interface is similar to WO_3_.^43^ In summary, the low-loss analysis confirms that a WO_3_ layer forms on top of the entire sapphire substrate when epitaxial WS_2_ growth is obtained.

Low-loss EELS analysis was also performed on the noEpi sample. In WS_2_-free areas, a clear interface layer cannot be identified. It is noted that strong beam damage of the WS_2_ layer is observed in the noEpi sample (see Figure S17(c)). Moreover, charging effects disturbed the imaging of the noEpi sample, which were not observed in the Epi sample. These observations indicate that there is a fundamental difference at the surface and interface of the sapphire substrate between epitaxially and nonepitaxially grown samples.

**Figure 5:**
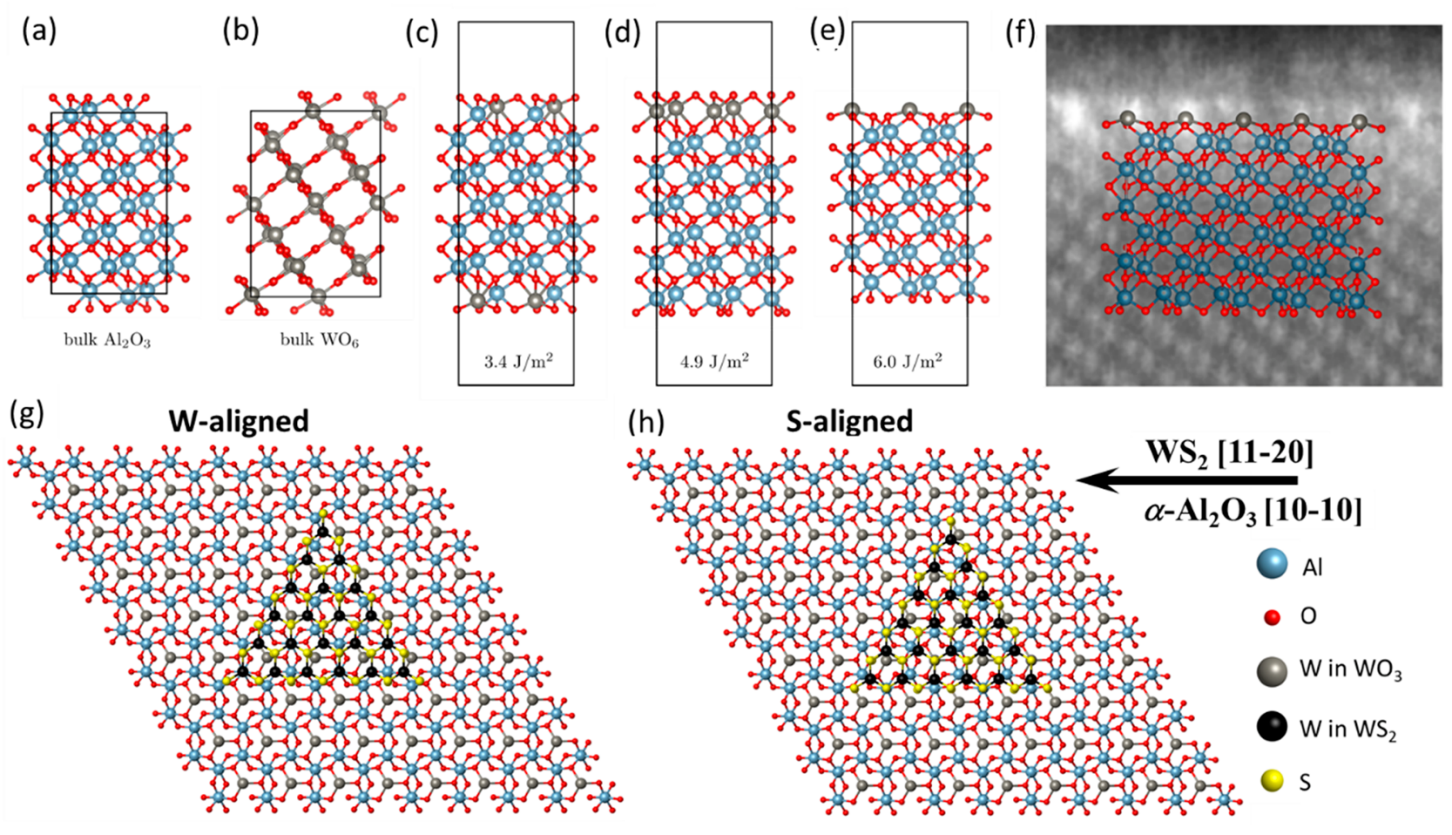
Results of DFT calculations. Schematic representation of bulk (a) Al_2_O_3_ and (b) WO_3_ crystals, and (c–e) possible WO_3_-modified sapphire surfaces with increasing separation energies. (f) Superposition of the structure in (e) on top of a section of one of the HRTEM images. (g,h) Schematic representation of the aligned WS_2_ domains on the WO_3_ terminated sapphire substrate for the W- and S-aligned cases, respectively.

To gain insight into the atomic structure of our materials, we carried out density-functional theory (DFT) calculations. As an initial step, we investigated the ground state structures of bulk alumina, Figure 5(a), and bulk tungsten oxide, Figure 5(b), obtaining results that agree with the previous literature (see the Supporting Information for more details on our methodology and results). We then simulated slabs of alumina with tungsten atoms deposited on them.

The atoms of (111) tungsten layers in bulk WO_3_ occupy very similar positions to the atoms of (0001) aluminum layers in bulk Al_2_O_3_. Both structures have cations that occupy octahedral sites formed from close packing of oxygen anions. Therefore, we optimized slabs of Al_2_O_3_ terminated by a layer of additional oxygen atoms and a layer of tungsten atoms on top and on bottom. The energetically optimal arrangement is displayed in Figure 5(c);^465^ the resulting structure can be seen as an alumina slab where its outer Al-layer atoms have been replaced by W atoms, keeping all ions in their preferred valence state.

Detailed inspection of some of the STEM images suggest that this kind of substitution might occur for an Al layer that is on top of an O layer (and not on top of another Al layer). To explore this possibility, we next substituted two, Figure 5(d), and one, Figure 5(e), Al layers into W layers on top of an oxygen layer. Figure 5(f) shows that the latter case agrees quite well with the results of STEM imaging. In these last two cases, the slabs are terminated differently on top and bottom to allow all ions to be Al^3+^, O^2–^, or W^6+^. To compare the energetics of these slabs with one of the previous symmetric slabs, we resorted to computing their separation energy: the energy cost to remove the WO_3_ or W_2_O_6_ layers from the Al_12_O_18_ alumina slab. The highest of these energies occurs for the configuration in Figure 5(e), in line with the good match shown in Figure 5(f).

Following the DFT calculations, the tungsten coverage on the WO_3_ terminated *c*-plane substrates was measured by X-ray fluorescence. The W coverage was determined to be 5.2 W/nm^2^, see Figure S20 for details, which fits very well the calculated tungsten surface density from the DFT derived structure (5.07 W/nm^2^). Hence, we conclude the WO_3_ structure presented in Figures 5(e,f), in which one W layer on top of an oxygen layer matching the structure of an Al layer in the α-Al_2_O_3_ crystal, is the most stable configuration matching the cross-section STEM image, Figure 5(f), and the W coverage measurements. Adhesion energies of WS_2_ on both bare and WO_3_-terminated α-Al_2_O_3_ slabs were calculated with two different high-symmetry alignments, labeled W- and S-aligned, schematically shown in Figures 5(g,h), respectively. The adhesion energies for WS_2_ on bare α-Al_2_O_3_ were calculated to be 70 meV/Å^2^ and 73 meV/Å^2^ for W-aligned and S-aligned WS_2_, respectively. The corresponding adhesion energies for WO_3_-terminated α-Al_2_O_3_ were 54 meV/Å^2^ and 53 meV/Å^2^. These results indicate that there is little energetic preference between the two alignments, but a fairly significant reduction in adhesive strength with the presence of the WO_3_ layer. This corresponds to stronger bonding between S and O in the bare sapphire compared with weaker bonding between the W and S in the WO_3_-terminated sapphire. Typical adhesion energies in layered van der Waals materials typically range between 10 and 30 meV/Å^2^.^47^

The results described above demonstrate that an interfacial continuous layer of WO_3_ is formed on the *c*-plane sapphire surface on epi-grown WS_2_ samples, and absent or less homogeneous in nonepitaxially grown cases. Hence, such a layer appears to provide another path for the Q-vdW epitaxial growth of WS_2_ on the basal plane of single-crystal alumina substrates. Taking this into account, previous reports on quasi-vdW epitaxial growth of TMDCs on sapphire were revisited with careful consideration. The outcome is shown in Table S1. In several reports, an intentional or unintentional metal seeding step is done prior to the growth. For example, an unintentional metal seeding using metal oxide (MoO_3_ or WO_3_) precursors might be achieved while heating the growth samples (and the metal oxide precursor) without the addition of the chalcogen. These oxides might be partially reduced while heating the reactor in a hydrogen atmosphere, and partial vapor pressures of a few mTorr could be achieved before the chalcogen supply.^21,24,29,31,36^ Such pressures are high enough to flow a substantial amount of precursor to the growth substrate, acting unintentionally as a metal-seeding step. Similarly, a metal-seeding in MOCVD was performed by increasing the metal–carbonyl precursor flow in the beginning of the growth.^12,25,35^ Such an excess in metal precursor leads to a high metal supersaturation level on the surface prior to the reaction with the chalcogen. One major difference in our procedure is the use of O_2_ or H_2_O vapors. At this stage, however, it is unclear if and how this plays a role in the WO_3_ formation and/or Q-vdW epitaxial growth. Some of the previous works cited above used O_2_ flows to avoid metal-oxide precursor spoiling during the growth process.^23^ XPS characterization is also reported in some of the cited works, showing the presence of the W(Mo)O_3_ (depending on the precursors used) phase, and usually attributed to some oxidation of the TMDC layer.^12,24,31,37^ In addition, as mentioned above, there are two epitaxial relationships reported between the TMDC and *c*-plane sapphire; [11–20]//[10–10] and [11–20]//[11–20], see Table S1. Such registry variations could point to a different interface chemistry. Further research is needed in order to fully understand the correlation between the surface chemical termination and the Q-vdW epitaxial growth. In this work, we hypothesize the metal and chalcogen precursor’s residence time and surface diffusion on the WO_3_ modified alumina is larger than on the bare α-Al_2_O_3_, therefore facilitating the oriented growth at the initial stages of the TMDC nucleation and growth.

In order to further demonstrate the robustness of the metal-seeding approach, two sets of growth experiments were performed. In the first, a W-seeding step was implemented by exposing the sapphire substrate only to the metal carbonyl precursor in an MOCVD reactor, as described above. No chalcogen was delivered to the growth substrate, see Experimental Section for details. Following the metal-seeding step, the substrates were cooled and transferred to a metal-oxide (MoO_3_) based CVD system for the growth of MoS_2_. As a result, quasi-vdW epitaxial MoS_2_ domains were obtained in a system never achieved before. In the second set of experiments, an intentional Mo-seeding step was performed in the same metal-oxide CVD system *prior* to the supply of the sulfur, achieved by preheating the MoO_3_ precursor and growth substrate, before the chalcogen. Once again, quasi-vdW epitaxial growth of MoS_2_ on sapphire was obtained. Figure S21 summarizes these results. Hence, W or Mo metal seeding step leads to similar MoS_2_ oriented growth on sapphire, demonstrating the robust effect the metal-seeding step has on the Q-vdW epitaxial growth of TMDCs in general on sapphire.

### Conclusions

A mechanism of quasi-vdW epitaxial growth of TMDCs on *c*-plane sapphire is revealed. The oriented growth is enabled by the formation of an ordered WO_3_ layer on top of the single-crystal alumina. Such well-ordered surface modification might be responsible for dictating the orientation of the initial TMDC nuclei on the surface, and thus facilitating the quasi-vdW epitaxial growth. WO_3_ is also detected on nonepitaxial samples, especially underneath the TMDC domains; however, it is not ordered and homogeneous as on samples in which the metal-seeding step was applied. This work shines light on the epitaxial formation of TMDCs on 3D substrates, by elucidating a mechanism for the Q-vdW epitaxial growth. This work might be the foundation to study the rational design of quasi-vdW epitaxial systems by a careful consideration of the modification of the growth surface, prior to the growth.

## Experimental Section

### MOCVD Growth

The synthesis of WS_2_ was carried out using a hot wall 3 in. customized MOCVD furnace (CVD equipment corporation, model Easy Tube 2000), equipped with 4 separate bubblers for precursors. In one bubbler, Di-*tert*-butyl sulfide (DTBS, sigma Aldrich, 97%) was loaded inside a glovebox under inert gas and the second bubbler was loaded with W(CO)_6_ (Strem Chemicals, 99.9%) in the same glovebox. The choice of other source of sulfur was H_2_S. For this, we used a scrubber system to neutralize the residual gas. The background and carrier gas used were argon (99.9999%) and hydrogen (99.999%). The substrates, *c*-plane sapphire (annealed at 1050 °C for 10 h) were cleaned using acetone and IPA each for 10 min in an ultrasonicator and were dried using a nitrogen gun. Prior to the growth, the furnace was evacuated to a pressure down to 15 mTorr for 15 min to remove any unwanted moisture/oxygen species. Thereafter, the furnace was ramped to 850 °C at a heating rate of 20 °C/min for 30 min under 50 Torr with 100 sccm of oxygen to remove any possibilities of carbon contamination. Then the oxygen flow was stopped and the system was maintained at the same (850 °C) temperature for another 25 min. All the required precursors were then released for the growth. We followed a growth and etch technique in our process cycle as schematically shown in Figures 2(a,b). The process cycle was mainly a combination of two main steps with a period of 5 min. In the first step, all the precursors (H_2_, DTBS, W(CO)_6_, H_2_O) were introduced for the growth to take place. In the second step, only H_2_O was introduced for the etching process. We repeated this sequence for 3–4 cycles, always terminating with the growth step. The hydrogen and H_2_O flow was varied from recipe to recipe. The amount of H_2_O introduced was measured by a residual gas analyzer (RGA), as described previously. The calculated flow of W(CO)_6_ and DTBS was found to be ∼3.28 × 10^–7^ mol/min and ∼3.25 × 10^–4^ mol/min, respectively, which leads to a sulfur to metal ratio of (S/M) ∼ 1000.

### Metal Seeding in MOCVD

Before the growth, the *c*-plane sapphire (preannealed at 1050 °C for 10 h) substrates were cleaned by ultrasonication in acetone and IPA (each for 10 min), followed by blow drying with a nitrogen gun. The growth of the WS_2_ monolayers was carried out at a temperature of 850 °C (pressure of 50 Torr) after in situ annealing in O_2_. The metal seeding technique was adopted to obtain epitaxial WS_2_ monolayer domains. Following the annealing in O_2_, tungsten hexacarbonyl was flown with a high concentration of ∼3.28 × 10^–7^ mol/min with argon background (no S precursor). Then the W(CO)_6_ was reduced to ∼1.31 × 10^–8^ mol/min with a DTBS flow of 3.25 × 10^–4^ mol/min, typically for additional 60 min. In addition to the metal and chalcogen sources, 5 mmol/min H_2_O (see SI for details), 25 sccm of H_2_, and 500 sccm of Ar were flown during the process. After the growth, the furnace was allowed to cool down naturally under an argon background.

### Metal Seeding in MOCVD (W) and M–O (Mo) and MoS_2_ Q-vdW Epitaxial Growth

At first, a c-sapphire substrate was annealed under an oxygen atmosphere for 30 min in a MOCVD system, and thereafter a metal seeding step was performed at 850 °C for 5 min and subsequently cooled to room temperature. Then the W-seeded substrate was transferred to a metal oxide (M–O, MoO_3_) based CVD system. In a typical growth process, 3 mg of MoO_3_ powder is placed in a ceramic boat and the W preseeded *c*-plane sapphire substrate was put facing upward in an upstream position on the same boat. In another boat, 350 mg of sulfur powder was taken downstream and was heated separately (150–200 °C) using an external heating tape. The growth was carried out at 720 °C 15 min. For the MoO_3_ seeding experiments, the experimental setup was the same, but prior to the heating of the sulfur source, the *c*-plane sapphire was exposed for 7 min only to the MoO_3_ precursor. This was followed for the same growth of 15 min described above. The whole process was carried out using a confined space growth technique as previously reported.

### X-ray Photoelectron Spectroscopy (XPS)

XPS measurements were performed using a ThermoScientific ESCALAB QXi. The samples were irradiated with a monochromatic Al Kα radiation. High resolution spectra were collected with a 20 eV pass energy and XPS parallel imaging was collected with a 100 eV pass energy. Spot size for both measurements was 650 μm in diameter. Image resolution is 5 μm.

### Transfer of WS_2_ to TEM Grids, Quartz, and SiO_2_ Substrates

The transfer of WS_2_ film from the growth substrate to a new desired substrate was carried out by using a previously reported polystyrene (PS) technique.^48^ In this process, 450 mg of PS (280,000 g/mol) was dissolved in 5 mL of toluene. The PS solution was then spin-coated (60 s at 3500 rpm) onto the as-grown WS_2_ layer on sapphire to make a thin film of PS. Thereafter, the sample was backed at 90 °C for 30 min and then at 120 °C for 10 min. The polymer/WS_2_ assembly was delaminated by allowing the water to penetrate in between sapphire and the assembly. The floating layer was then fished out of the water to any desired substrate (TEM grid, quartz, or SiO_2_) and left for drying naturally at room temperature. Another baking process (90 °C for 30 min and more 15 min at 120 °C) was adopted to ensure no water residuals were left in the interface. In the end, the PS film was dissolved from the assembly by using toluene.

### HRSTEM, EDS, and EELS Measurements

High-resolution scanning transmission electron microscopy (HRSTEM) investigations have been conducted in a probe-corrected Titan (Thermo Fisher Scientific) with a high-brightness gun operated at 300 keV and a convergence angle of 25 mrad. Collection angle was 48 mrad for high-angle annular (HAA)DF imaging. An Oxford Instruments Ultim X-MaxN 100TLE detector was used for energy-dispersive X-ray spectroscopy (EDS) analysis and a Gatan Image Filter (GIF) Tridiem ESR 866 spectrometer for electron energy-loss spectroscopy (EELS) measurements. EELS collection angle was 52 mrad for W–M edge and 20 mrad for O–K edge and low-loss analysis. Standard k factors were used for EDX quantification using Aztec Software (Oxford Instruments). A custom Matlab-based software was used for the analysis of the EELS spectrum-image (SI) data, which was obtained of the low-loss area (including the W–N edge at 36 eV and Al–L at 73 eV) as well as of the O–K (532 eV), Al–K (1560 eV), and W–M edge (1809 eV). The low-loss spectra were treated by non-negative matrix factorization (NNMF) to elucidate the different contributions from protection layer, WS_2_, sapphire and the interface region. Prior to NNMF, we calibrated the energy axis using the zero-loss peak and selected an energy window from 2 to 50 eV as the inclusion of the intense zero-loss peak strongly affects the NNMF process. NNMF was performed several times on each SI data set using various factor numbers and consistency was checked with raw data. Samples were prepared by focused ion beam (FIB) assisted TEM lamella fabrication using a Helios 600 dual-beam instrument (Thermo Fisher Scientific) selecting a random area in the epitaxially grown sample, an area with two triangular WS_2_ flakes including a WS_2_-free area in the nonepitaxially grown sample and an area with aligned nanowire in the sample with long metal-seeding step (30 min). The specimens were coated with an amorphous carbon protection layer prior to lamella fabrication.

### Atomic Force Microscopy (AFM)

Topographic characterization was performed using a Nanosurf Core AFM in tapping mode.

### Raman and Photoluminescence Spectroscopy

The Raman and PL spectroscopy measurements were conducted using a confocal micro-Raman (PL) spectrometer (Horiba, LabRAM HR Evolution). The as-grown samples were excited using an excitation laser of 532 nm and collected the scattered radiation using ×100 objective (0.9 NA). The scattered radiation was analyzed and detected using a grating of 1800 g mm^–1^ and thermoelectrically cooled CCD detector, respectively. The Raman and PL spectral imaging were performed on a software controlled XYZ motorized stage with a step resolution of ∼100 nm. The laser power, exposure, and collection timings were optimized to record the Raman and PL imaging.

### In-Plane Grazing Incidence X-ray Diffraction

In-plane grazing incidence X-ray diffraction measurements used a 9 kW Cu anode Rigaku SmartLab with a 5-axis diffractometer. The incident beam from the horizontal line source was conditioned by a multilayer parabolic mirror, followed by a 0.5° horizontal soller slit, followed by a 0.05 mm-high by 5 mm-wide slit. The incident beam angle on the sample was fixed at α = 0.5°. The detector arm had a 0.5° horizontal soller slit with a 20 by 20 mm opening and ending with a Rigaku HyPix 2D detector operated in 0D mode, such that the out-of-plane component of the scattering angle β was integrated over the range of 0 to 2.5°. The λ = 1.542 Å incident Cu Kα flux on the sample was 3.9 × 10^7^ photons per second. In-plane reciprocal lattice points of the film and substrate were accessed by rotating the azimuthal ϕ angle of the sample and the in-plane 2θχ angle of the detector. The soller slits produced a 2θχ measured resolution of 0.39°. See Figures S10–S12 for further details.

### X-ray Fluorescence Spectroscopy

Using an 18 kW Mo anode Rigaku X-ray source with a Huber 2-circle diffractometer and Vortex silicon drift diode X-ray fluorescence detector, the W coverages were determined for the WO_3_ coated sapphire and WS_2_/WO_3_/sapphire samples to be 5.2 and 10.6 W/nm^2^, respectively. The incident beam from the vertical line source was conditioned by a parabolic multilayer mirror followed by a 0.2 mm-wide by 2.5 mm high slit. The λ = 0.711 Å incident Mo Kα flux on the sample was 7.9 × 10^7^ photons per second. The incident angle on the sample was set at α = 6.3°. The absolute coverage was determined by comparison to an RBS calibrated standard sample. The Be window of the 50 mm^2^ XRF detector was parallel to the sample surface and 10 mm away. Figure S13 shows the X-ray fluorescence spectrum.

### Density Functional Theory Calculations for WO_*x*_ Separation Energies

All our DFT calculations were carried out using the VASP software. To include the role of core electrons, we used the projected-augmented wave method, treating as valence electrons the 3*s* and 3*p* of Al, the 2*s* and 2*p* of O, and the 5*s*, 5*p*, 5*d*, and 6*s* of W. VASP uses plane waves to solve the Kohn–Sham equations;^2^ in our case we included those up to a kinetic-energy cutoff of 600 eV. Integrations in reciprocal space were carried out using grids that guaranteed that the smallest allowed spacing between nearest points in the grid is below 0.3 Å^–1^.

We used the exchange-correlation functional of Perdew, Burke, and Ernzerhof adapted to solids (PBEsol), which predicted bulk lattice parameters for alumina and tungsten oxide well within 1% difference with respect to experimental values. For all calculations involving slabs, we took the in-plane lattice parameters from the ones we computed for bulk alumina, and we made sure that enough vacuum space was added out-of-plane. All the atomic positions in our slabs were optimized until the forces on them were below 0.02 eV/ Å; the final in-plane stress components were in all cases below 5 GPa. The separation energy was computed by subtracting from a slab with alumina and tungsten oxide the energies of independent alumina and tungsten oxide sets of layers and dividing by the number of pairs of surfaces created and the in-plane area of their unit cell. Our tests showed that these energies vary less than 1% when the vacuum length of the cell is increased by 50%. For the slabs that contained a dipole moment, we applied the corresponding corrections to the calculation of the energies.

### Density Functional Theory Calculations for WS_2_ Adhesion Energies

Density functional theory calculations were carried out with the Vienna Ab-initio Simulation Package (VASP).^49,50^ All calculations were carried out using the Perdew–Burke–Ernzerhof (PBE) formalism of the generalized gradient approximation.^51^ The Monkhorst–Pack method was used to generate *k*-point meshes.^52^ The number of *k*-point divisions along direction *i*, *N*_*i*_, was chosen such that *N*_i_ × *a*_*i*_ ≈ 25 Å for geometric optimizations and ∼75 Å for PBE static calculations, with *a*_*i*_ being the length of the supercell. The kinetic energy cutoff for the plane-wave basis was 500 eV to ensure converged results. Geometric optimization was performed with a force convergence criterion of 10^–2^ eV/Å on all atoms. The atomic positions of the alumina slabs were relaxed while fixing in-plane lattice parameters to their bulk values. To get adhesion energy values, calculations on alumina slabs with symmetric WS_2_ on each side were performed, allowing only the atomic positions of the WS_2_ and the outer layers of Al_2_O_3_ and WO_3_ to be relaxed. The formula for the adhesion energy was *E*_adhesion_ = (*E*_slab+WS_2__ – 4*E*_slab_ – 18*E*_WS_2__)/2*A*, where *E*_slab+WS_2__ is the total energy of the slab with two adhered layers of WS_2_, *E*_slab_ is the total energy of the bare slab, and *E*_WS_2__ is the energy of free-standing WS_2_. *A* is the in-plane area of the slab + WS_2_ supercell. The factor of 4 corresponds to the 2 × 2 supercell of alumina, the factor of 18 corresponds to two 3 × 3 supercells of WS_2_, and the factor of 2 corresponds to the two sheets of WS_2_ on either surface. These calculations were performed with and without the WO_3_ layer and with two different high-symmetry alignments of the WS_2_ sheets with the alumina corresponding to some of the aluminum columns being coincident with W atoms or S atoms in the WS_2_.

## Supporting Information Available

Schematic representation of the MOCVD system, additional images of the aligned growth, XRD-setup and measurement description, XPS spectra and imaging data, CS STEM DF images of WO_3_ terminated sapphire, results from different metal-seeding and growth steps, extensive EELS measurements on Epi and noEpi samples, DFT calculation description, comparison table with previous reports on Q-vdW epitaxial growth, X-ray fluorescence data, and metal-seeding and Q-vdW epitaxial growth of MoS_2_ (PDF)
