# Supplementary material for: Tungsten Oxide Mediated Quasi-van der Waals Epitaxy of WS2 on Sapphire
Source: ACS Nano. 2023 Mar 8;17(6):5399–411. doi: 10.1021/acsnano.2c09754 (PMC10062024; doi:10.1021/acsnano.2c09754)
Supplement: Supplementary file 1 — nn2c09754_si_001.pdf [file nn2c09754_si_001.pdf]

Supporting Information

Tungsten Oxide Mediated Quasi - van der Waals Epitaxy of WS_2 on Sapphire

Assael Cohen,[1#] Pranab K. Mohapatra,[1#] Simon Hettler,[2,3] Avinash Patsha,[1] Narayanachari K.V.L.V.,[5] Pini Shekhter,[4] John Cavin,[5] James M. Rondinelli,[5] Michael Bedzyk,[5,6] Oswaldo Dieguez,[1] Raul Arenal[2,3,7] and Ariel Ismach[1*]

[1]Department of Materials Science and Engineering, Tel Aviv University, Ramat Aviv, Tel Aviv, 6997801, Israel

[2] Laboratorio de Microscopías Avanzadas (LMA), Universidad de Zaragoza, 50018 Zaragoza, Spain.

[3] Instituto de Nanociencia y Materiales de Aragón (INMA), CSIC-Universidad de Zaragoza, 50009 Zaragoza, Spain

[4] Center for Nanoscience and Nanotechnology, Tel Aviv University, Tel Aviv, 6997801, Israel

[5] Department of Materials Science and Engineering and [6]Department of Physics and Astronomy, Northwestern University, Evanston, Illinois 60208, United States

[7] ARAID Foundation, 50018 Zaragoza, Spain.

[#] These authors contributed equally to the work

* aismach@tauex.tau.ac.il

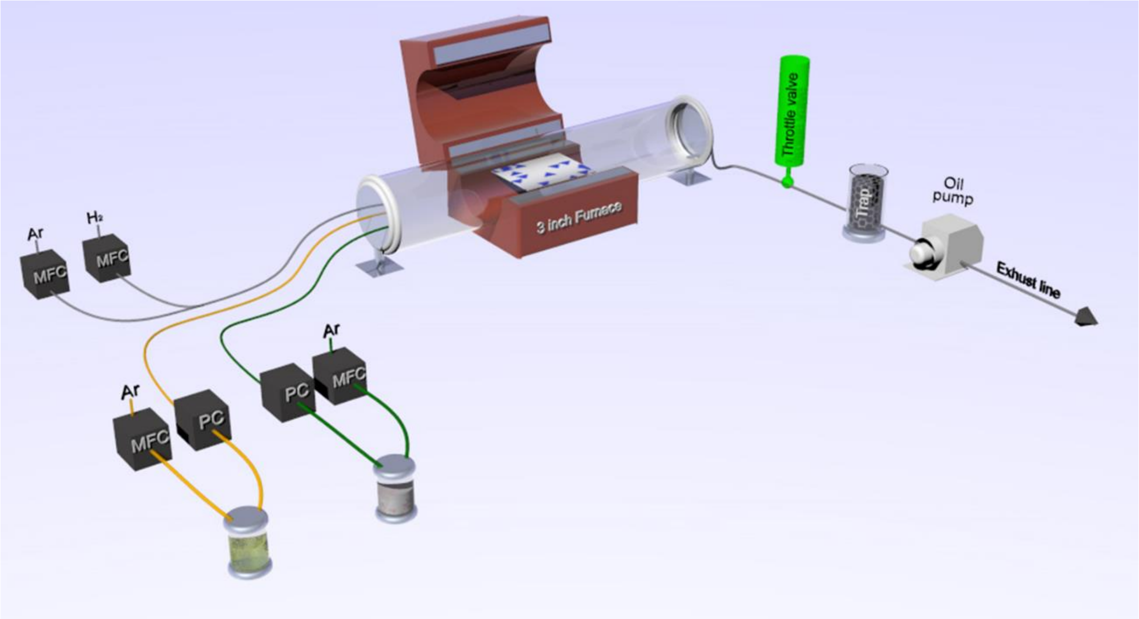

Figure S1: Schematic representation of the MOCVD system.

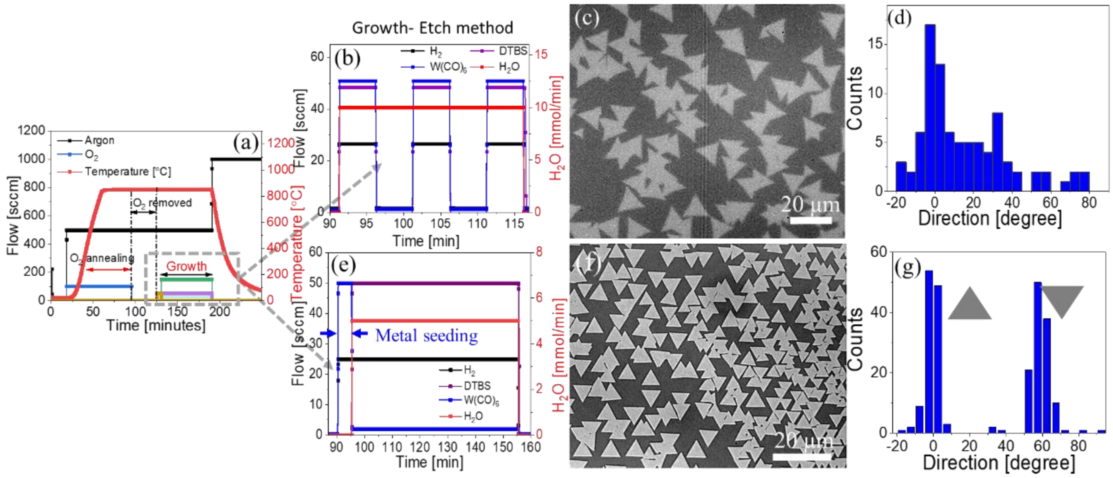

Figure S2: (a) General gas flow scheme. (b)-(d) Growth-etch method and results, showing an optical micrograph and the respective domain orientation distribution. (e)-(g) Metal-seeding approach and its results, exhibiting a directed growth, as seen in the optical microscope image, (f), and in the orientation distribution analysis, (g).

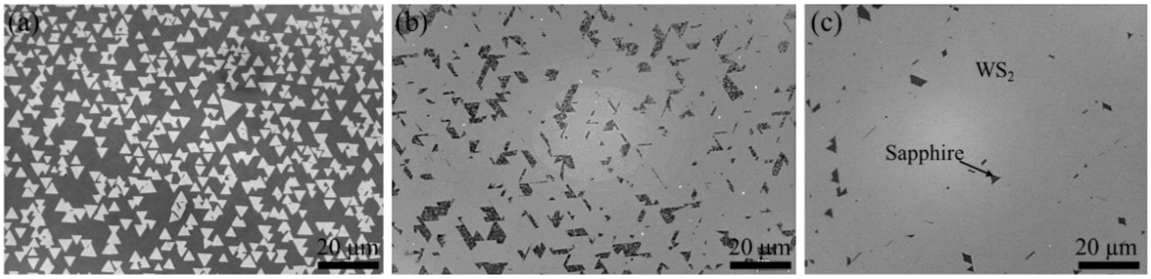

Figure S3: Quasi-vdW Epitaxial growth of WS_2 on sapphire. (a) – (c) Optical images with increasing coverage.

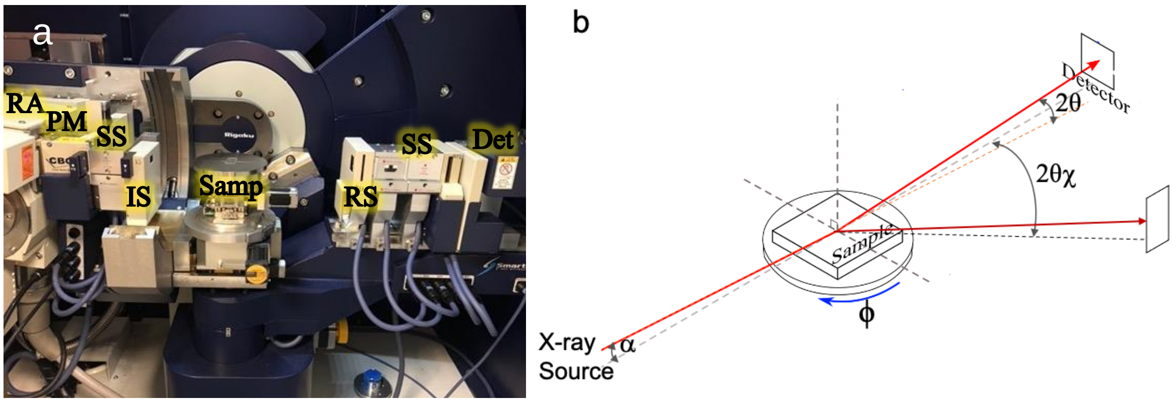

Figure S4: In-plane XRD data was collected on a Rigaku 9 kW SmartLab 5-circle diffractometer. a) RA: Cu Rotating Anode X-ray line source, PM: Parabolic multilayer collimating mirror, SS: 0.5° soller slits, IS: 0.05 x 5 mm incident slit, Sample, RS: open Receiving Slit, and 2D detector. b) Description of grazing incidence geometry with $\alpha = 2\theta =$ 0.5° fixed and variable angles ϕ and $2\theta\chi$.

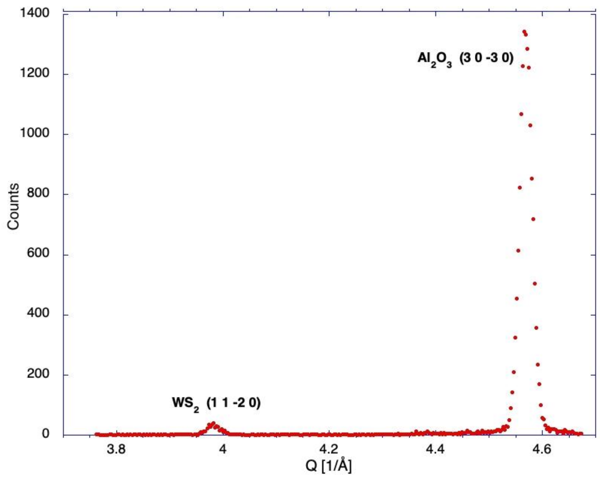

Figure S5: In-plane XRD $2\theta\chi-\phi$ radial scan along Al_2O_3 [3 0 -3 0] direction.

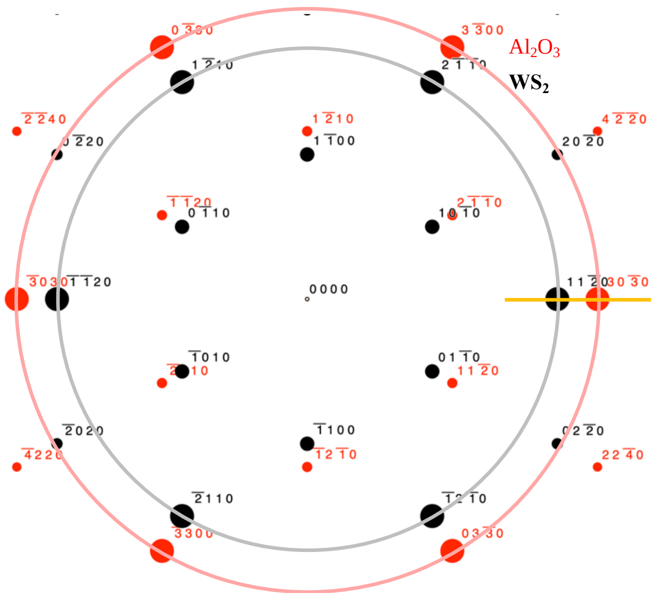

Figure S6: Overlapping bulk-like L = 0 layers in reciprocal space for WS_2 (black spots) and Al_2O_3 (red spots). The ϕ scans in Figure 1(g) followed the gray and light red circular paths centered at 0000 and passing through the family of {11-20} WS_2 and {30-30} Al_2O_3 reciprocal lattice points, respectively. The radial scan in Fig. S11 followed the yellow line.

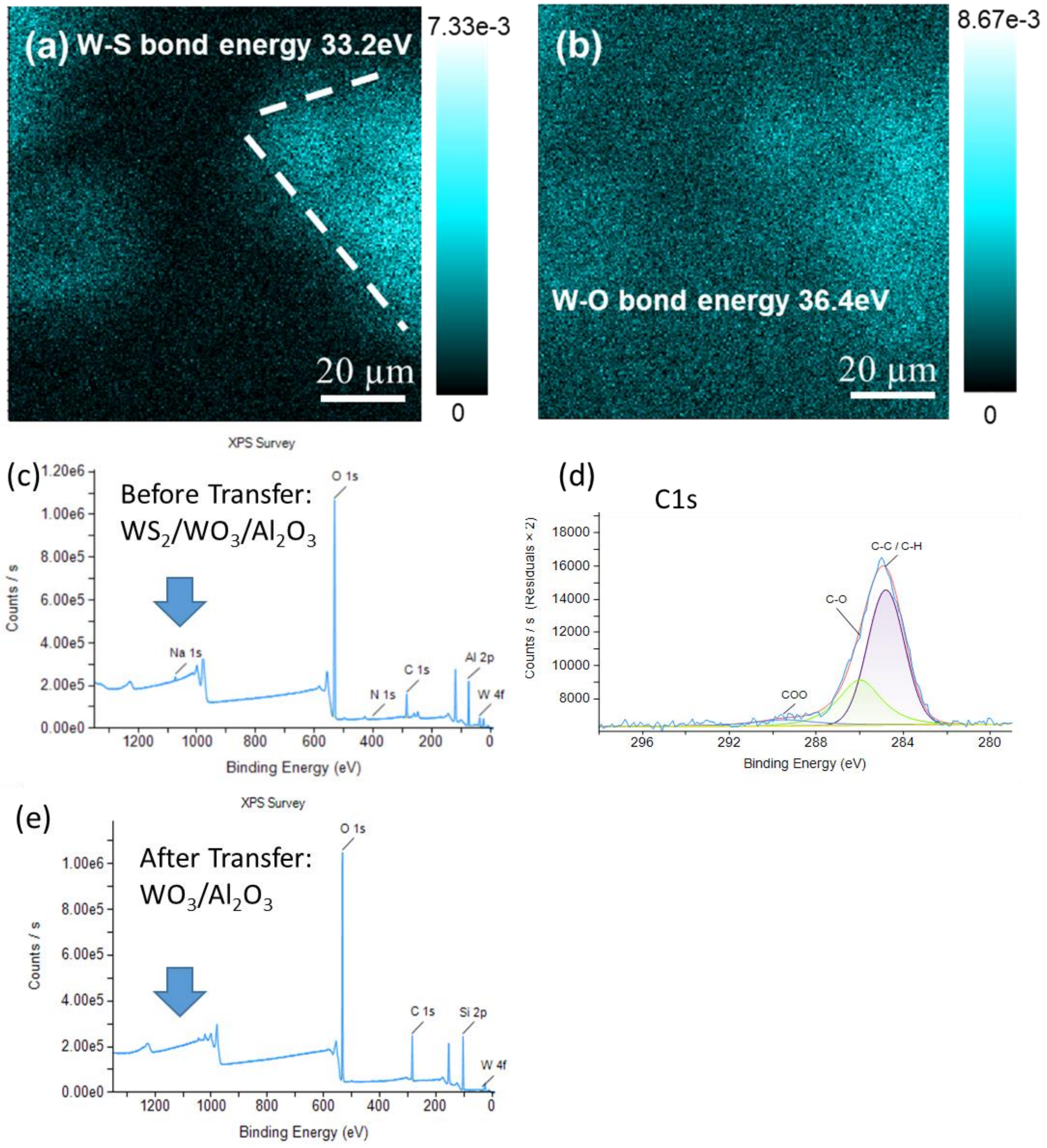

Figure S7: XPS characterization of a Q-vdW epitaxial grown WS_2 sample. (a)-(b) XPS imaging using the WS_2 and WO_3 peaks, respectively. The white dashed lines in (a) mark the edges of several WS_2 domain edges, light blue, and the WS_2-free area, black. The W-O bonds are detected across the entire surface of the sample. (c) XPS survey spectra of the same sample, showing some Na (very small signal) and C presence. (d) High resolution C1s scan showing the presence of adventitious carbon. (e) XPS survey after the transfer of the WS_2 domains, showing the Na is no longer detected.

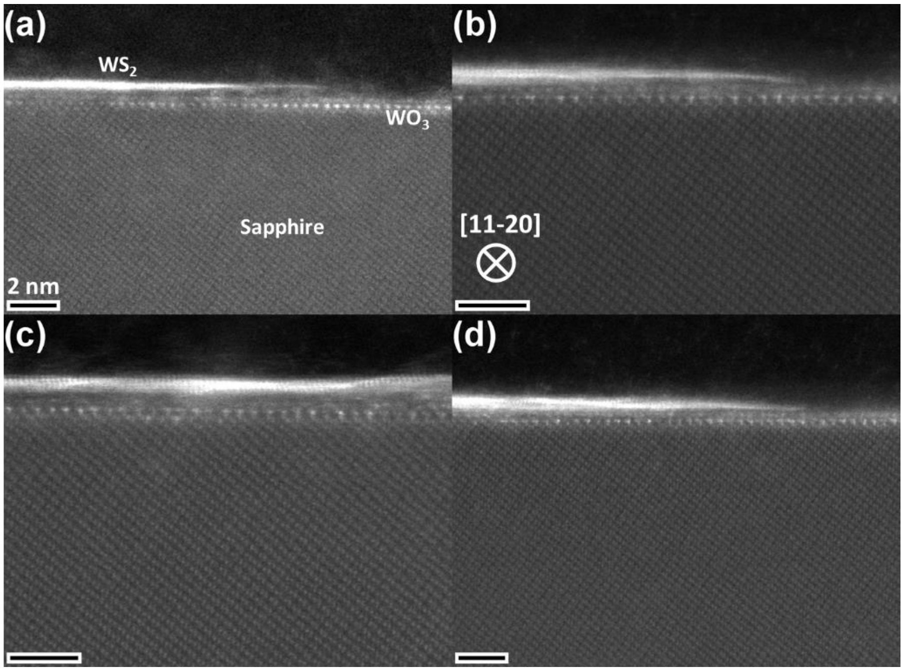

Figure S8: Additional HAADF STEM cross section images of the epi growth samples in which the WS_2 (upper brighter layer), the WO_3 (ordered on the sapphire surface) and the sapphire (bulk-bottom, the [11-20] direction towards the paper).

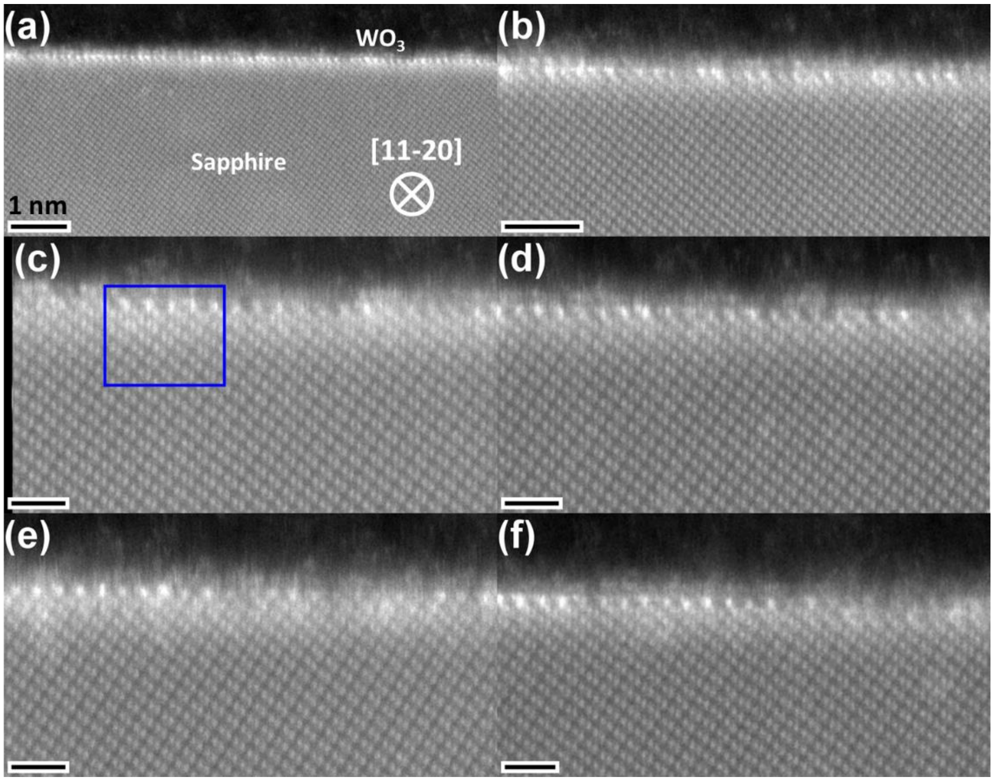

Figure S9: Additional HAADF STEM cross section images of the epi growth samples in WS_2 – free areas. The WO_3 (ordered on the sapphire surface) and the sapphire (bulk-bottom, the [11-20] direction towards the paper).

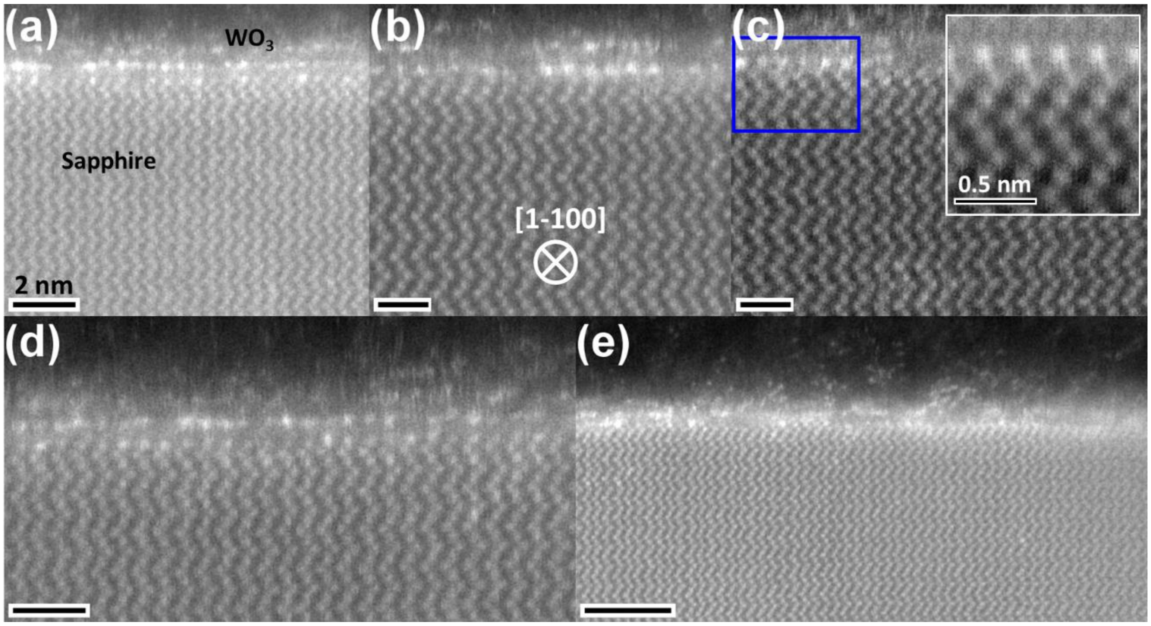

Figure S10: Additional HAADF STEM cross section images of the epi growth samples in WS_2 – free areas. The WO_3 (ordered on the sapphire surface) and the sapphire (bulk-bottom, the [1-100] direction towards the paper). The inset in (c) shows the atomic arrangement at the interface, which we obtained from averaging several unit cells of a high-resolution image.

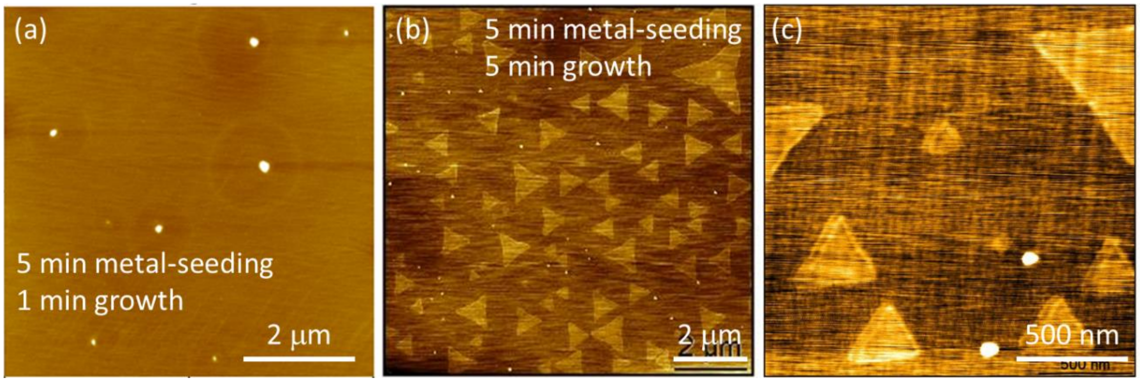

Figure S11: AFM characterization of samples after different growth times (a) AFM image showing particles after a 1 min growth (after 5 mins. of metal seeding). (b)-(c) AFM images showing the results for a 5 mins. metal-seeding and 5 mins. growth. Aligned relatively small domains together with nanoparticles are clearly seen.

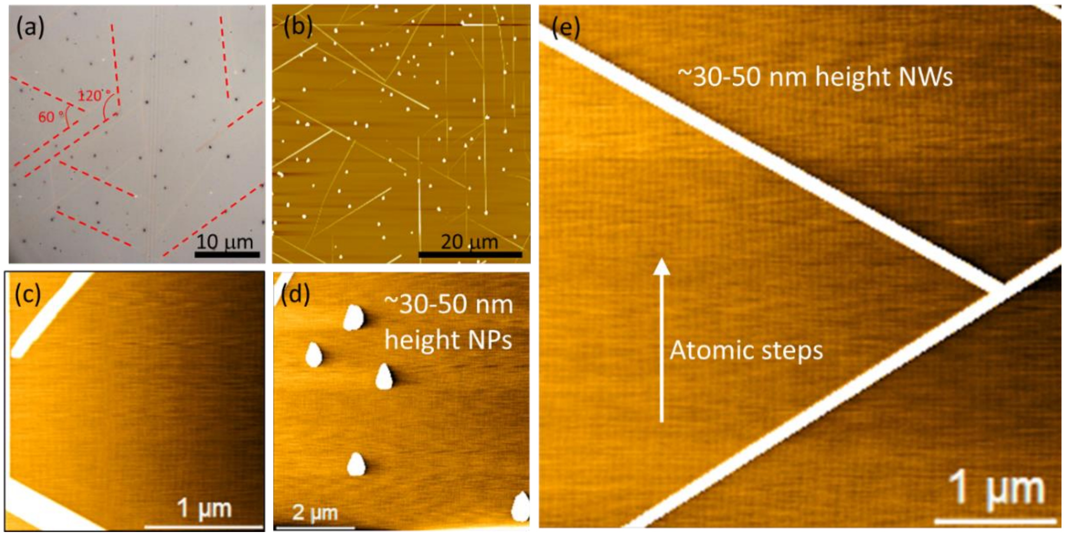

Figure S12: Long metal-seeding step, 30 mins. (a) Optical microscope image showing aligned nanowires and particles on the growth substrate, sapphire. (b)-(e) AFM images showing the aligned NWs and particles.

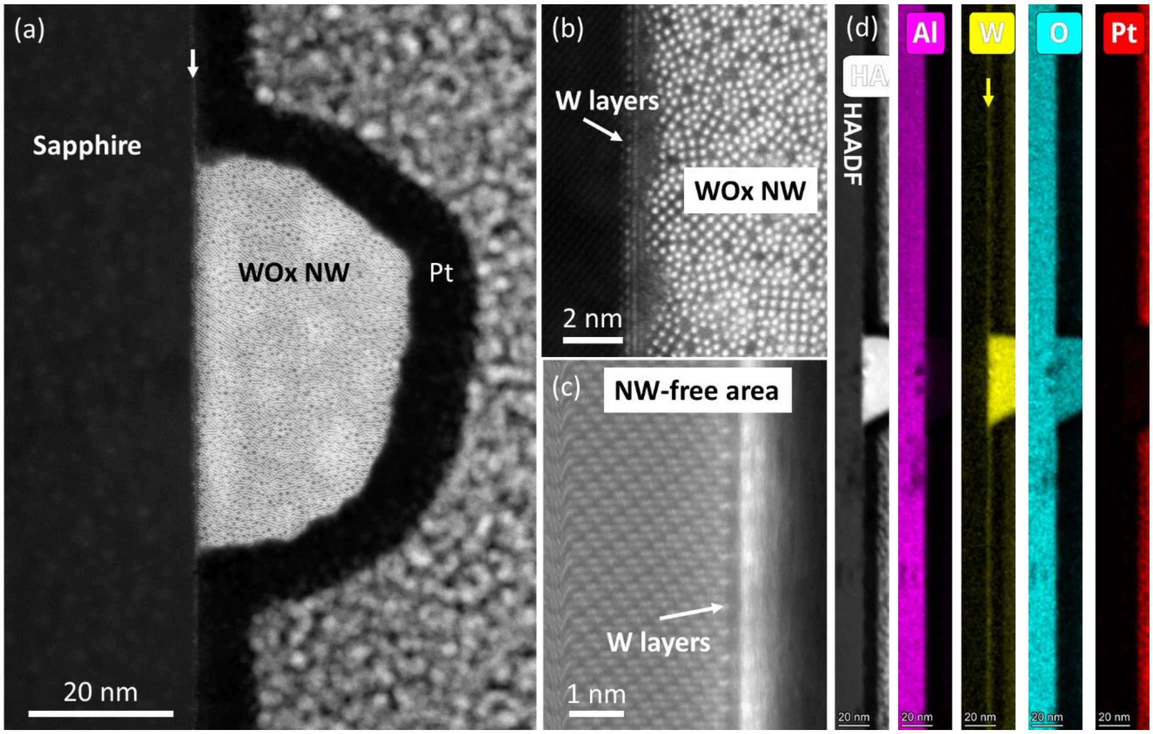

Figure S13: Long metal-seeding step, 30 mins: Cross section STEM analysis. (a) Low magnification CS STEM HAADF image of a NW. The white arrow indicates the high contrast sapphire surface termination, attributed to the presence of W. (b)-(c) High resolution HAADF images at the NW-sapphire interface, (b), and on a NW-free area, (c). In both cases high contrast atoms can be observed at the sapphire surface with the same structure. At the interface with the NW, few well-ordered W atomic layers are seen (1-4), after that the arrangement seems to break into a more disordered structure, (b). (d) EDS analysis, from left to right, HAADF image, elemental mapping with Al, W, O and Pt. Again the yellow arrow in the W mapping shows the presence of tungsten in the NW-free areas.

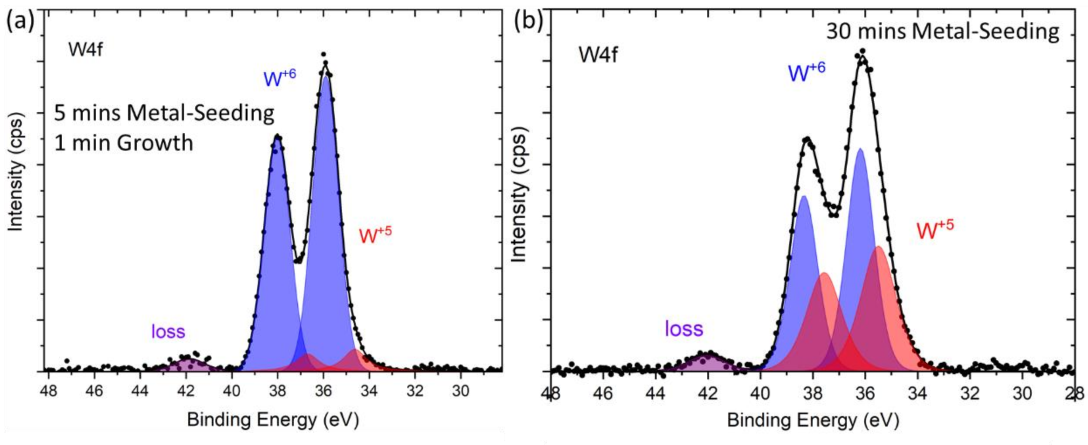

Figure S14: XPS characterization of a sample after 5 mins metal seeding and 1 min growth, (a), and after 30 mins metal seeding, (b). In (a) a small tungsten sub-oxide is seen (in red) while its contribution is much pronounced after 30 mins metal seeding, (b). The reason for the latter probably being the presence of the amorphous nanowires which have mixed oxide stoichiometry.

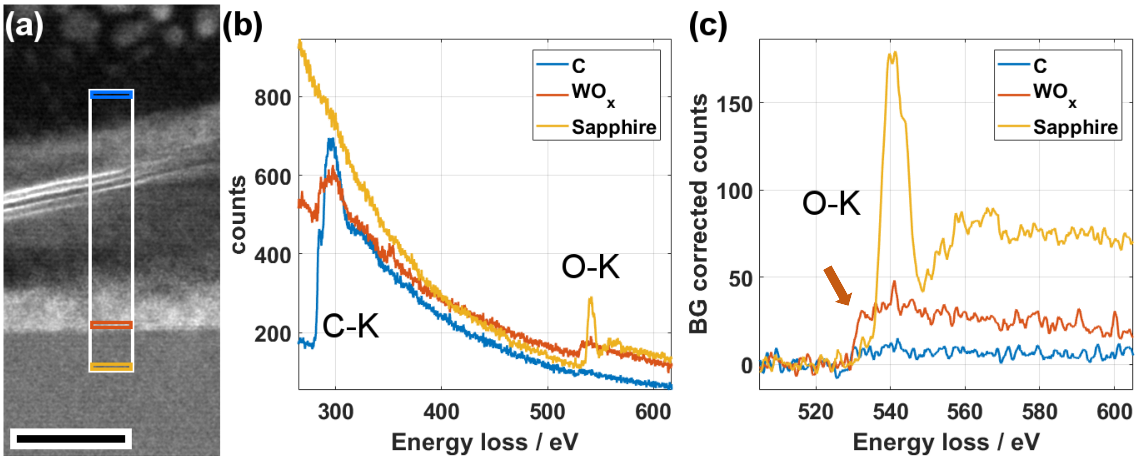

Figure S15: EELS analysis of small contamination particle in noEpi sample. (a) STEM-DF image with investigated area marked by a white frame and areas selected for spectra in (b) and (c) marked by colored rectangles. (b) EEL spectra reveal C-K edge at 284 eV and O-K at 530 eV. A small peak around 350 eV in interface region (WOx) is attributed to Ca. (c) Onset of O-K edge is shifted to lower energy in interface region (WOx). Scale bar is 9 nm.

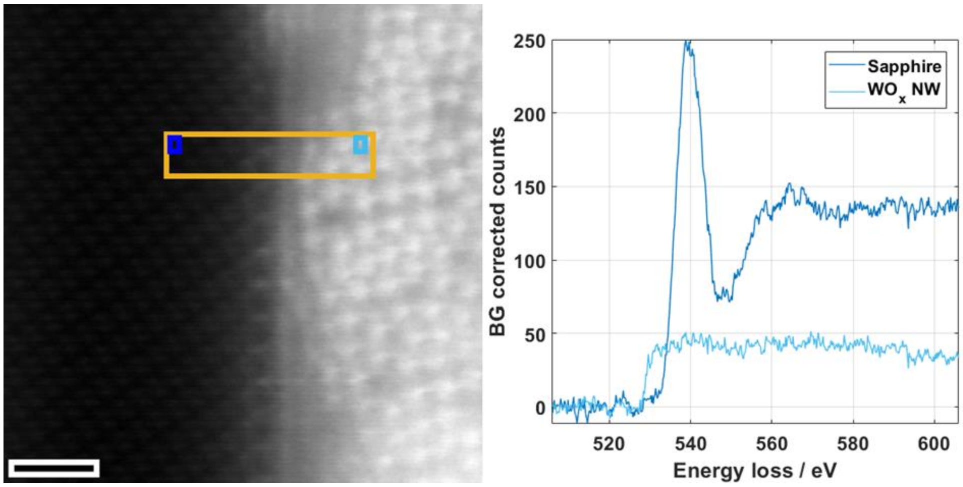

Figure S16: EELS analysis of a WOx NW after 30 mins. Metal-seeding. Left, STEM-DF image with the marked areas for the EELS spectra, right. It can be seen that the WOx NW exhibits a flat curve after the early onset and no main peak.

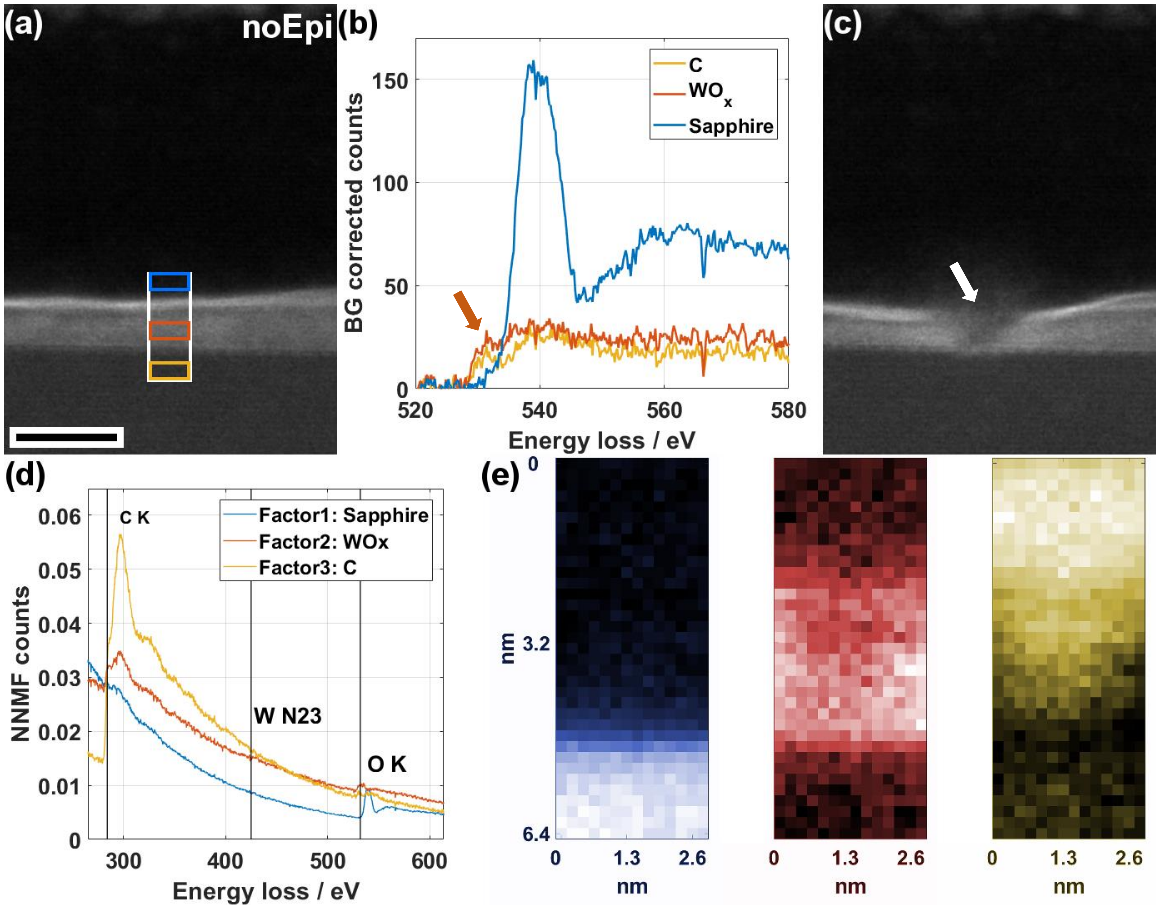

Figure S17: STEM-EELS analysis of interface region between sapphire and WS_2. (a) STEM-DF image with investigated area marked by a white frame. (b) Background-subtracted spectra from regions marked in (a) show again shift of O-K onset energy to

lower energies in interface region (WO_x). (c) STEM-DF image taken after acquisition of the EELS data shows that the WS_2 layer is completely damaged, white arrow. (d) NNMF leads to three factors, which are attributed to sapphire substrate, interface region (WO_x) and carbon protection layer. A factor for WS_2 is not found due to the rapid damaging. The spectra reveal the C-K and O-K edge and a small peak at the W-$N_{2,3}$ edge position is observed in the interface region. (e) Spatial distribution of different factors agree well with expectation from the image. Scale bar is 7 nm.

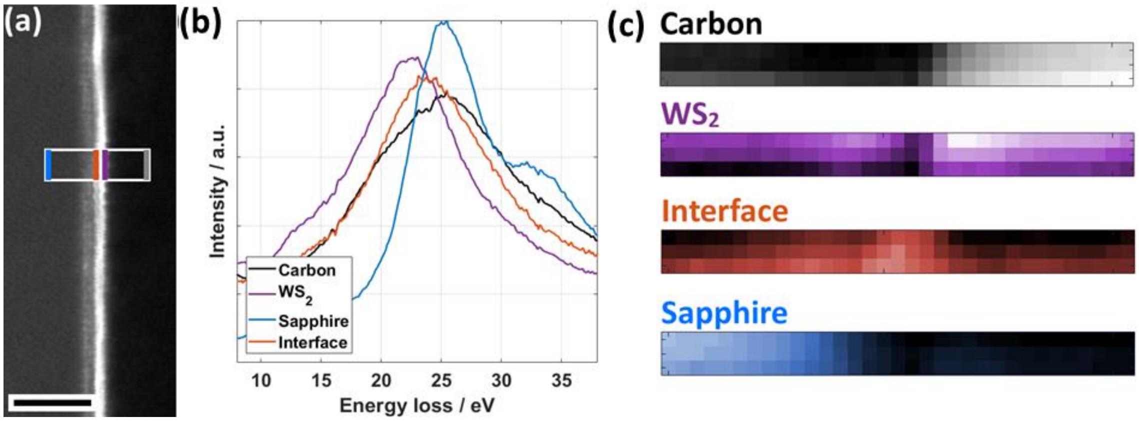

Figure S18: Low-loss EELS analysis of Epi sample in an area with WS_2. (a) STEM-DF image of investigated area (white rectangle) showing from left to right sapphire substrate, interface, WS_2 and carbon protection layer. (b) Four factors resulting from NNMF analysis of the EELS data can be assigned to contributions from carbon (black), sapphire (blue), interface (red) and WS_2 (purple). (c) Spatial distribution of factors agrees well with expectation from imaging. Minimum of WS_2 at position of actual WS_2 layer is explained by the strong scattering by the W atoms and an overall decrease of intensity in the EEL spectra. Scale bar is 4 nm.

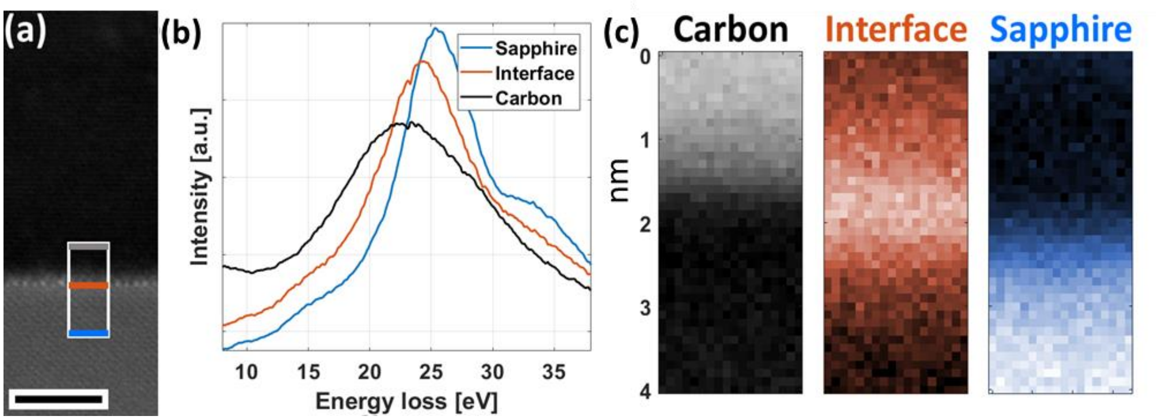

Figure S19: Low-loss EELS analysis of Epi sample in an area without WS_2. (a) STEM-DF image of investigated area (white rectangle) showing from bottom to top sapphire substrate, interface and carbon protection layer. (b) Three factors resulting from NNMF analysis of

the EELS data can be assigned to contributions from carbon (black), sapphire (blue) and interface (red). (c) Spatial distribution of factors agrees well with expectation from imaging. Scale bar is 4 nm.

Density-Functional Theory Calculations

Our density-functional theory (DFT) calculations were carried out using the VASP software.[1] Our basis set included plane waves with a kinetic energy cutoff of 600 eV. The electrons were treated within the projector-augmented wave method;[2] we explicitly treated the following as valence electrons: 2s and 2p of oxygen; 3s and 3p of aluminum; and 5d, 6s, and 6p of tungsten. We used the revised Perdew-Burke-Ernzerhof functional for solids (PBEsol)[3] to handle exchange and correlation effects.

Optimization of bulk alumina resulted in hexagonal lattice parameters of a = 4.773 Å and c = 13.008 Å, in agreement with previous DFT calculations and experimental results (in all our optimization calculations the resulting forces were below 0.02 eV/Å). Bulk tungsten oxide can crystallize in a variety of distorted perovskite phases; we performed structural optimizations starting from each of the 20 unit cells available at the Open Quantum Materials Database (OQMD)[4] and we obtained similar results to those in a recent study of the polymorphism of this material:[5] the most stable phase has P-1 symmetry, and it is, in our case, 86 meV per formula unit below the simple-cubic phase. A comparison of the metal atoms in the relaxed structures shows that while in Al_2O_3 the Al atoms of a layer occupy the sites of an hexagonal two-dimensional lattice of parameter 4.773 Å, in WO_3 the W atoms of a layer are displaced from the hexagonal lattice points by up to tenths of an Å, so that first neighbors are between 5.083 Å and 5.677 Å apart.

We proceeded to simulate slabs of Al_2O_3 with W atoms deposited on them by taking a unit cell like the one of Figure 5(a) and allowing for vacuum at the top and bottom of it (we used the same 6 layers of oxygen and 12 layers of aluminum of the bulk, but we used a simulation cell with a c parameter at least twice as large as the bulk one to account for that vacuum next to the surfaces). On top (and, to keep symmetry, on bottom) of this symmetric aluminum-terminated slab we added one layer of W and one layer of O. For the tungsten layer, we tried the two possible arrangements of hexagonal symmetry compatible with the aluminum layers (one that continues the same stacking pattern, and one that creates a stacking fault). Regarding the oxygen layer added, we tried the two orientations present in alumina, and the two orientations present in WO_3 along [111]. We tried stacking both the W layer on top of the aluminum, and the oxygen layer on top of the aluminum. In all, we started with 16 possible configurations, which after atomic relaxation (keeping the lattice vectors constant) evolved to 6 inequivalent configurations. The lowest-energy configuration among these is oxygen terminated, and its outermost metal layer contains full W occupancy; it is the one represented in Figure 5(c) of the main text. The rest of slab configurations described in the main text were done following a similar procedure, but correcting the energy results for the presence of a surface dipole when needed. To compute the separation energies, we subtracted from the energy of the Al_2O_3 slab with deposited

tungsten the energy of the isolated Al_2O_3 slab and the energy of the adequate isolated set of layers of tungsten and oxygen.

Table S1: “Metal-seeding” step in previous reports on Q-vdW epitaxial growth.

**Title**	**Method**	**Metal seeding**	**Orientation (TMDC ‖ SAPPHIRE)**	**Ref.**
Diffusion-Controlled Epitaxy of Large Area Coalesced WSe_2 Monolayers on Sapphire	Cold-wall MOCVD	High flow of metal precursor in the early stages of the growth	[11-20] ‖ [11-20]	6
Wafer-Scale Epitaxial Growth of Unidirectional WS_2 Monolayers on Sapphire	Cold-wall MOCVD	High flow of metal precursor in the early stages of the growth	[11-20] ‖ [11-20]	7
Substrate Lattice-Guided MoS_2 Crystal Growth: Implications for van der Waals Epitaxy	*In-situ* M-O CVD	Growth at 790 °C, flow O_2 at 500 °C for the in-situ oxidation/ evaporation of a Mo foil for 10 min with no Sulfur.	**[11-20] ‖ [10-10]**	8
Suppressing Nucleation in Metal–Organic Chemical Vapor Deposition of MoS_2 Monolayers by Alkali Metal Halides	Hot-wall MOCVD	Metal-seeding step at 600 °C for 30 mins. Heated to the growth temperature (800–1050 °C) and only then the S precursor, H_2S, was introduced.	Not specified	9
Epitaxial Growth of highly-aligned MoS_2 on C-Plane Sapphire	M-O CVD	Growth at 650 °C, the sulfur was heated only when reactor temperature was ~600 °C	[11-20] ‖ [11-20]	10
Large-Area Epitaxial Monolayer MoS_2	M-O CVD	Growth at 700 °C, the sulfur was heated only when reactor temperature was ~600 °C	[11-20] ‖ [11-20]	11
Substrate Lattice-Guided Seed Formation Controls the Orientation of 2D Transition-Metal Dichalcogenides	M-O CVD	Growth at 800 °C, the sulfur was heated only when reactor temperature was ~500 °C	**[11-20] ‖ [10-10]** And [11-20] ‖ [11-20]	12
Epitaxial growth and interfacial property of monolayer MoS_2 on gallium nitride	M-O CVD	Growth at 800 °C, the sulfur was heated only when reactor temperature was ~500 °C	Gallium nitride substrate	13

Hydrogen-assisted step-edge nucleation of $MoSe_2$ monolayers on sapphire substrates	M-O CVD	Growth at 800 °C. Simultaneous heating of the MoO_3 and Se at different heating rates, in which the metal oxide reaches ~500 °C and more before the calchogen is effectively evaporated.	**[11-20] ǁ [10-10]**	14
Realizing Large-Scale, Electronic-Grade Two-Dimensional Semiconductors	M-O CVD	High flow of metal precursor in the early stages of the growth	[11-20] ǁ [11-20]	15
Monolayer MoS_2 on sapphire: an azimuthal reflection high-energy electron diffraction perspective	Cold-wall MOCVD	Annealing in H_2 at 1000 °C before the growth it itself. and the growth also in 1000°C	[11-20] ǁ [11-20]	16
Step engineering for nucleation and domain orientation control in WSe_2 epitaxy on c-plane sapphire	Cold-wall MOCVD	Metal-seeding step at 850-850 °C. Heated to the growth temperature (650–1050 °C), H_2Se flow during all processes and cooling till 300 °C.	[11-20] ǁ [11-20]	17
Dual-coupling-guided epitaxial growth of wafer-scale single-crystal WS_2 monolayer on vicinal a-plane sapphire	M-O CVD	Not specified	A plane sapphire substrate	18
Epitaxial growth of wafer-scale molybdenum disulfide semiconductor single crystals on sapphire	M-O CVD	Growth at 850–1000 °C , high sulfur flow of 300 sccm of Ar, also 3 sscm flow of O_2 for the MoO_3 (could lead to passivation layer)<br><br>Not specified	**[11-20] ǁ [10-10]**	19
Surface-Mediated Aligned Growth of Monolayer MoS2 and In-Plane Heterostructures with Graphene on Sapphire	M-O CVD	Studied the S supply influence on the growth	**Intermediate S supply [11-20] ǁ [10-10]**<br>High S supply [11-20] ǁ [11-20]	20
Step-Edge-Guided Nucleation and Growth of Aligned WSe2 on Sapphire via a Layer-over-Layer Growth Mode	M-O CVD	Step-edge guided growth	[11-20] ǁ [11-20]	21
M-O CVD: Metal Oxide (MoO_3, WO_3, etc.) Chemical Vapor Deposition				
MOCVD: Metal Organic Chemical Vapor Deposition				

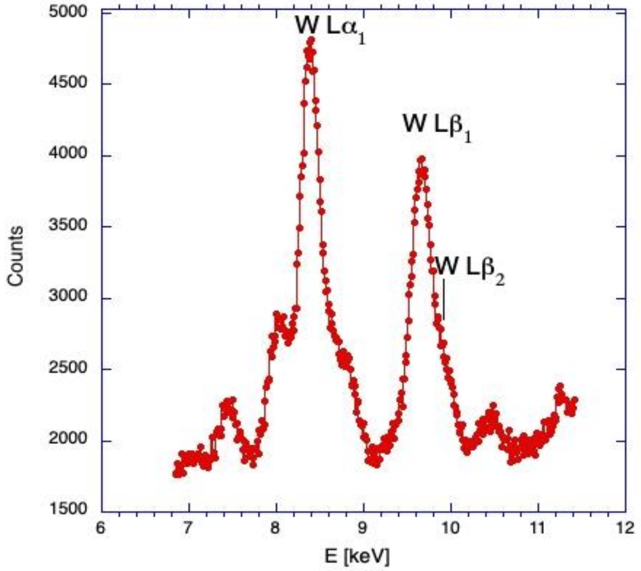

Figure S20: X-ray fluorescence spectrum collected in 2000 s in the vicinity of the W L lines from a $WS_2/WO_3/\ Al_2O_3(0001)$ sample using 17.44 Kev X-rays with an incident intensity of $8x10^7$ photons /s and at a 6° incident angle. The 50 mm^2 Si drift-diode XRF detector was facing the sample and 10 mm away from the 20 mm^2 radiated spot. Under identical conditions, spectra were collected from the WO_3 sample and a Si/Ge/Si quantum well calibrated standard. The count rates were corrected for background counts and detector dead-time. To convert XRF cps into atoms per nm^2 we used the 17.44 keV relative XRF cross section for Ge Kα of 2891 and W $L\beta_1$ of 1753. These calculated values are based on an extension of Puri *et al.* [22 22 22] The WS_2-free sample had 5.2 W / nm^2, the WS_2/WO_3/sapphire sample had 10.6 W/ nm^2. This would imply 5.4 W/ nm^2 in the WS_2 layer, which is equivalent to 0.5 ML of WS_2.

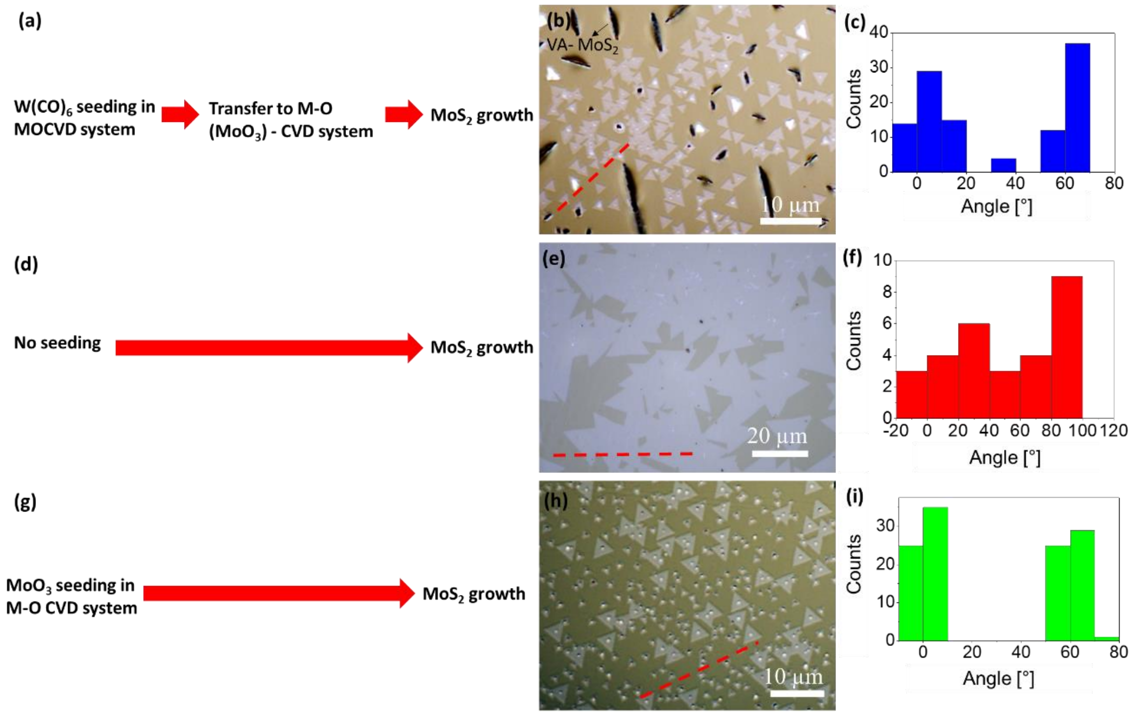

Figure S21: Metal seeding Q-vdW epitaxy of MoS_2 in a metal-oxide (MoO_3) based CVD system. Tungsten carbonyl seeding in MOCVD system, transfer to the MO_3 based CVD and growth of Q-vdW epitaxy of MoS_2. (b) No metal-seeding growth and (c), MoO_3 based metal seeding. (d)-(f) Respective optical images showing the oriented growth when the metal seeding using $W(CO)_6$, (d), or MoO_3, (f), is implemented. (c)-(i) Orientation histograms for the above samples. The dashed red line in the optical micrographs represent the angle=0°.

References

(1) Kresse, G.; Hafner, J. ABINITIO MOLECULAR-DYNAMICS FOR LIQUID-METALS. *Physical Review B* **1993**, *47* (1), 558-561. DOI: 10.1103/PhysRevB.47.558. Kresse, G.; Hafner, J. AB-INITIO MOLECULAR-DYNAMICS SIMULATION OF THE LIQUID-METAL AMORPHOUS-SEMICONDUCTOR TRANSITION IN GERMANIUM. *Physical Review B* **1994**, *49* (20), 14251-14269. DOI: 10.1103/PhysRevB.49.14251. Kresse, G.; Furthmuller, J. Efficiency of ab-initio total energy calculations for metals and semiconductors using a plane-wave basis set. *Computational Materials Science* **1996**, *6* (1), 15-50. DOI: 10.1016/0927-0256(96)00008-0. Kresse, G.; Furthmuller, J. Efficient iterative schemes for ab initio total-energy calculations using a plane-wave basis set. *Physical Review B* **1996**, *54* (16), 11169-11186. DOI: 10.1103/PhysRevB.54.11169.

(2) Blöchl, P. E. Projector augmented-wave method. *Physical Review B* **1994**, *50* (24), 17953-17979. DOI: 10.1103/PhysRevB.50.17953. Kresse, G.; Joubert, D. From ultrasoft pseudopotentials to the projector augmented-wave method. *Physical Review B* **1999**, *59* (3), 1758-1775. DOI: 10.1103/PhysRevB.59.1758.

(3) Perdew, J. P.; Ruzsinszky, A.; Csonka, G. I.; Vydrov, O. A.; Scuseria, G. E.; Constantin, L. A.; Zhou, X. L.; Burke, K. Restoring the density-gradient expansion for exchange in solids and surfaces. *Physical Review Letters* **2008**, *100* (13). DOI: 10.1103/PhysRevLett.100.136406.

(4) Saal, J. E.; Kirklin, S.; Aykol, M.; Meredig, B.; Wolverton, C. Materials Design and Discovery with High-Throughput Density Functional Theory: The Open Quantum Materials Database (OQMD). *Jom* **2013**, *65* (11), 1501-1509. DOI: 10.1007/s11837-013-0755-4.
(5) Hamdi, H.; Salje, E. K. H.; Ghosez, P.; Bousquet, E. First-principles reinvestigation of bulk ${\mathrm{WO}}_{3}$. *Physical Review B* **2016**, *94* (24), 245124. DOI: 10.1103/PhysRevB.94.245124.
(6) Zhang, X.; Choudhury, T. H.; Chubarov, M.; Xiang, Y.; Jariwala, B.; Zhang, F.; Alem, N.; Wang, G.-C.; Robinson, J. A.; Redwing, J. M. Diffusion-Controlled Epitaxy of Large Area Coalesced WSe2 Monolayers on Sapphire. *Nano Letters* **2018**, *18* (2), 1049-1056. DOI: 10.1021/acs.nanolett.7b04521.
(7) Chubarov, M.; Choudhury, T. H.; Hickey, D. R.; Bachu, S.; Zhang, T.; Sebastian, A.; Bansal, A.; Zhu, H.; Trainor, N.; Das, S.; et al. Wafer-Scale Epitaxial Growth of Unidirectional WS2 Monolayers on Sapphire. *ACS Nano* **2021**, *15* (2), 2532-2541. DOI: 10.1021/acsnano.0c06750.
(8) Lai, Y.-Y.; Chuang, C.-H.; Yeh, Y.-W.; Hou, C.-H.; Hsu, S.-C.; Chou, Y.; Chou, Y.-C.; Kuo, H.-C.; Wu, Y. S.; Cheng, Y.-J. Substrate Lattice-Guided MoS2 Crystal Growth: Implications for van der Waals Epitaxy. *ACS Applied Nano Materials* **2021**, *4* (5), 4930-4938. DOI: 10.1021/acsanm.1c00469.
(9) Kim, H.; Ovchinnikov, D.; Deiana, D.; Unuchek, D.; Kis, A. Suppressing Nucleation in Metal–Organic Chemical Vapor Deposition of MoS2 Monolayers by Alkali Metal Halides. *Nano Letters* **2017**, *17* (8), 5056-5063. DOI: 10.1021/acs.nanolett.7b02311.
(10) Kang, L.; Tian, D.; Meng, L.; Du, M.; Yan, W.; Meng, Z.; Li, X.-a. Epitaxial growth of highly-aligned MoS2 on c-plane sapphire. *Surface Science* **2022**, *720*, 122046. DOI: https://doi.org/10.1016/j.susc.2022.122046.
(11) Dumcenco, D.; Ovchinnikov, D.; Marinov, K.; Lazić, P.; Gibertini, M.; Marzari, N.; Sanchez, O. L.; Kung, Y.-C.; Krasnozhon, D.; Chen, M.-W.; et al. Large-Area Epitaxial Monolayer MoS2. *ACS Nano* **2015**, *9* (4), 4611-4620. DOI: 10.1021/acsnano.5b01281.
(12) Aljarb, A.; Cao, Z.; Tang, H.-L.; Huang, J.-K.; Li, M.; Hu, W.; Cavallo, L.; Li, L.-J. Substrate Lattice-Guided Seed Formation Controls the Orientation of 2D Transition-Metal Dichalcogenides. *ACS Nano* **2017**, *11* (9), 9215-9222. DOI: 10.1021/acsnano.7b04323.
(13) Yan, P.; Tian, Q.; Yang, G.; Weng, Y.; Zhang, Y.; Wang, J.; Xie, F.; Lu, N. Epitaxial growth and interfacial property of monolayer MoS2 on gallium nitride. *RSC Advances* **2018**, *8* (58), 33193-33197, 10.1039/C8RA04821E. DOI: 10.1039/C8RA04821E.
(14) Hwang, Y.; Shin, N. Hydrogen-assisted step-edge nucleation of MoSe2 monolayers on sapphire substrates. *Nanoscale* **2019**, *11* (16), 7701-7709, 10.1039/C8NR10315A. DOI: 10.1039/C8NR10315A.
(15) Lin, Y.-C.; Jariwala, B.; Bersch, B. M.; Xu, K.; Nie, Y.; Wang, B.; Eichfeld, S. M.; Zhang, X.; Choudhury, T. H.; Pan, Y.; et al. Realizing Large-Scale, Electronic-Grade Two-Dimensional Semiconductors. *ACS Nano* **2018**, *12* (2), 965-975. DOI: 10.1021/acsnano.7b07059.
(16) Xiang, Y.; Sun, X.; Valdman, L.; Zhang, F.; Choudhury, T. H.; Chubarov, M.; Robinson, J. A.; Redwing, J. M.; Terrones, M.; Ma, Y.; et al. Monolayer MoS2 on sapphire: an azimuthal reflection high-energy electron diffraction perspective. *2d Materials* **2021**, *8* (2). DOI: 10.1088/2053-1583/abce08.
(17) Zhu, H.; nayir, N.; Choudhury, T.; Bansal, A.; Huet, B.; Zhang, K.; Puretzky, A.; Bachu, S.; York, K.; Knight, T. M.; et al. Step engineering for nucleation and domain orientation control in WSe2 epitaxy on c-plane sapphire. Research Square: 2022.
(18) Wang, J.; Xu, X.; Cheng, T.; Gu, L.; Qiao, R.; Liang, Z.; Ding, D.; Hong, H.; Zheng, P.; Zhang, Z.; et al. Dual-coupling-guided epitaxial growth of wafer-scale single-crystal WS2 monolayer on vicinal a-plane sapphire. *Nature Nanotechnology* **2022**, *17* (1), 33-38. DOI: 10.1038/s41565-021-01004-0.

(19) Li, T.; Guo, W.; Ma, L.; Li, W.; Yu, Z.; Han, Z.; Gao, S.; Liu, L.; Fan, D.; Wang, Z.; et al. Epitaxial growth of wafer-scale molybdenum disulfide semiconductor single crystals on sapphire. *Nature Nanotechnology* **2021**, *16* (11), 1201-1207. DOI: 10.1038/s41565-021-00963-8.
(20) Suenaga, K.; Ji, H. G.; Lin, Y.-C.; Vincent, T.; Maruyama, M.; Aji, A. S.; Shiratsuchi, Y.; Ding, D.; Kawahara, K.; Okada, S.; et al. Surface-Mediated Aligned Growth of Monolayer MoS2 and In-Plane Heterostructures with Graphene on Sapphire. *ACS Nano* **2018**, *12* (10), 10032-10044. DOI: 10.1021/acsnano.8b04612.
(21) Chen, L.; Liu, B.; Ge, M.; Ma, Y.; Abbas, A. N.; Zhou, C. Step-Edge-Guided Nucleation and Growth of Aligned WSe2 on Sapphire via a Layer-over-Layer Growth Mode. *ACS Nano* **2015**, *9* (8), 8368-8375. DOI: 10.1021/acsnano.5b03043.
(22) Puri, S.; Chand, B.; Mehta, D.; Garg, M. L.; Singh, N.; Trehan, P. N. K and L Shell X-Ray Fluorescence Cross Sections. *Atomic Data and Nuclear Data Tables* **1995**, *61* (2), 289-311. DOI: https://doi.org/10.1006/adnd.1995.1012.
